# Functional characterization and transcriptional activity analysis of *Dryopteris fragrans* farnesyl diphosphate synthase genes

**DOI:** 10.3389/fpls.2023.1105240

**Published:** 2023-03-24

**Authors:** Dongrui Zhang, Xun Tang, Lingling Chen, Xiaojie Qiu, Chunhua Song, Hemeng Wang, Ying Chang

**Affiliations:** ^1^ College of Life Sciences, Northeast Agricultural University, Harbin 150030, China; ^2^ MOA Key Laboratory of Crop Ecophysiology and Farming System in the Middle Reaches of the Yangtze River, College of Plant Science & Technology , Huazhong Agricultural University, Wuhan 430070, China

**Keywords:** *Dryopteris fragrans*, farnesyl diphosphate synthase, terpenoid metabolic, gene function analyses, transcriptional activity analyses

## Abstract

Farnesyl diphosphate synthase (FPS), a key enzyme of the terpene metabolic pathway, catalyzes the precursor of sesquiterpene compounds farnesyl diphosphate (FPP) synthesis, and plays an important role in regulating plant growth and development. *Dryopteris fragrans* is a medicinal plant rich terpenoids. In this study, the function of the gene was verified *in vitro* and *in vivo*, the promoter of the gene was amplified and its transcriptional activity was analyzed. In the present study, we report the molecular cloning and functional characterization of *DfFPS1* and *DfFPS2*, two *FPS* genes from *D. fragrans*. We found that the two genes were evolutionarily conserved. Both *DfFPS* genes were highly expressed in the gametophyte and mature sporophyte leaves, and their expression levels increased in response to methyl jasmonate (MeJA) and high temperature. Both DfFPS proteins were localized in the cytoplasm and could catalyze FPP synthesis *in vitro*. We also found that the overexpression of *DfFPS* genes in tobacco plants promoted secondary metabolite accumulation but exhibited negligible effect on plant growth and development. However, the transgenic plants exhibited tolerance to high temperature and drought. The promoters of the two genes were amplified using fusion primer and nested integrated polymerase chain reaction (FPNI-PCR). The promoter sequences were truncated and their activity was examined using the β-glucuronidase (*GUS*) gene reporter system in tobacco leaves, and we found that both genes were expressed in the stomata. The transcriptional activity of the promoters was found to be similar to the expression pattern of the genes, and the transcriptional core regions of the two genes were mainly between −943 bp and −740 bp of *proDfFPS1*. Therefore, we present a preliminary study on the function and transcriptional activity of the *FPS* genes of *D. fragrans* and provide a basis for the regulation of terpene metabolism in *D. fragrans*. The results also provide a novel basis for the elucidation of terpene metabolic pathways in ferns.

## Introduction

Farnesyl diphosphate synthase (FPS; EC 2.5.1.10) is a major chain elongation enzyme of the isoprenoid pathway, which belongs to the *E*-family of prenyltransferases (Dhar et al., 2013). FPS catalyzes the condensation of dimethylallyl diphosphate (DMAPP; C5) with isopentenyl diphosphate (IPP; C5) in a head-to-tail way to form geranyl diphosphate (GPP; C10), resulting in a 10-C compound, which further condenses with another molecule of IPP to form the final product FPP (C15) (Dhar et al., 2013). FPS is a key enzyme of the branching point of the isoprene metabolic pathway, and the FPP produced by FPS is an important intermediate of the isoprenoid pathway, which can be used as the precursor for sesquiterpenoids, phytosterols, triterpenoids, abscisic acid (ABA), plastoquinone, coenzyme Q, and farnesylated proteins. The first plant *FPS* gene was cloned from *Arabidopsis thaliana* (Delourme et al., 1994); subsequently *FPS* genes were cloned from *Nicotiana tabacum* (Devarenne et al., 1998), *Oryza sativa* (Sanmiya et al., 1997), and other model plants. Most plant species have two *FPS* genes, *Arabidopsis* has two *FPS* genes that exhibit some degree of functional redundancy and act complementarily in seed development (Keim et al., 2012) and the *Arabidopsis fps1 fps2* double homozygous mutant exhibits embryo lethality, suggesting that the lack of this enzyme affects plant development during early stages (Closa et al., 2010). The specific signaling module in laticifer cells of *Hevea brasiliensis* (COI1-JAZ3-MYC2) can activate the expression of *FPS* to enhance rubber biosynthesis (Banyai et al., 2010). Since the recent development and utilization of traditional Chinese medicine, *FPS*, a key enzyme of the terpenoid metabolic pathway of various medicinal plants, has gained attention. For example, the overexpression of *FPS* in *Artemisia annua* can significantly increase artemisinin content and the fresh weight of its leaves (Banyai et al., 2010). Therefore, the investigation of the function of *FPS* in medicinal plants can lay a foundation for the biosynthesis of terpenoids and enhance the use of traditional Chinese medicine.

*Dryopteris fragrans* (L.) Schott, a member of the Dryopteridaceae family, is mainly distributed in the temperate regions of North America, Europe, and Asia; it is mostly distributed in the northeast part of China (Chen et al., 2020). *D. fragrans* is a fern with glandular trichomes and is rich in secondary metabolites, including phloroglucinols, flavonoids, lignans, terpenes, and other phenolic derivatives, such as coumarin, which are responsible for its biological activities, such as antibacterial, anti-inflammatory, antioxidant, and anti-tumor activities (Zhang et al., 2018). Several bioactive terpenoids have been reported from *D. fragrans*; however, the genes related to its terpenoid metabolic pathway have not been elucidated. A previous study revealed that squalene synthase (SQS) is the key gene of the triterpenoid biosynthesis pathway (Gao et al., 2019). Sesquiterpenes are volatile terpenes, and their precursor is FPP, which is synthesized by FPS. The expression and activity of FPS affects the production of sesquiterpenoids in plants.

To verify the function and activity of the *FPS* genes from *D. fragrans* (*DfFPS*), we analyzed and cloned the genes based on the transcriptome data of *D. fragrans*. First, we examined the conserved sequences of the two genes and predicted two proteins that can produce FPP. The expression pattern of the genes in different tissues and under hormonal and abiotic stresses was investigated using quantitative polymerase chain reaction (qPCR). Their localization in *Arabidopsis* protoplasts was analyzed, and their enzymatic properties were investigated *in vitro*. To confirm their function *in vivo*, transgenic tobacco plants were developed, and we found that the terpene content of the transgenic plants increased. To the best of our knowledge, for the first time, we report that the transgenic plants acquired some degree of resistance against drought and heat stress. To investigate the effects of the environment on the genes are affected by the environment, we cloned the promoters of the genes and analyzed tissue-specific expression tissue and transcriptional activity. We transiently transformed the tobacco leaves to obtain the transcriptional core regions of both genes. This study will provide insight for subsequent research on FPS.

## Results

### Molecular cloning and sequence analyses of *DfFPS1* and *DfFPS2*

**Figure 1:**
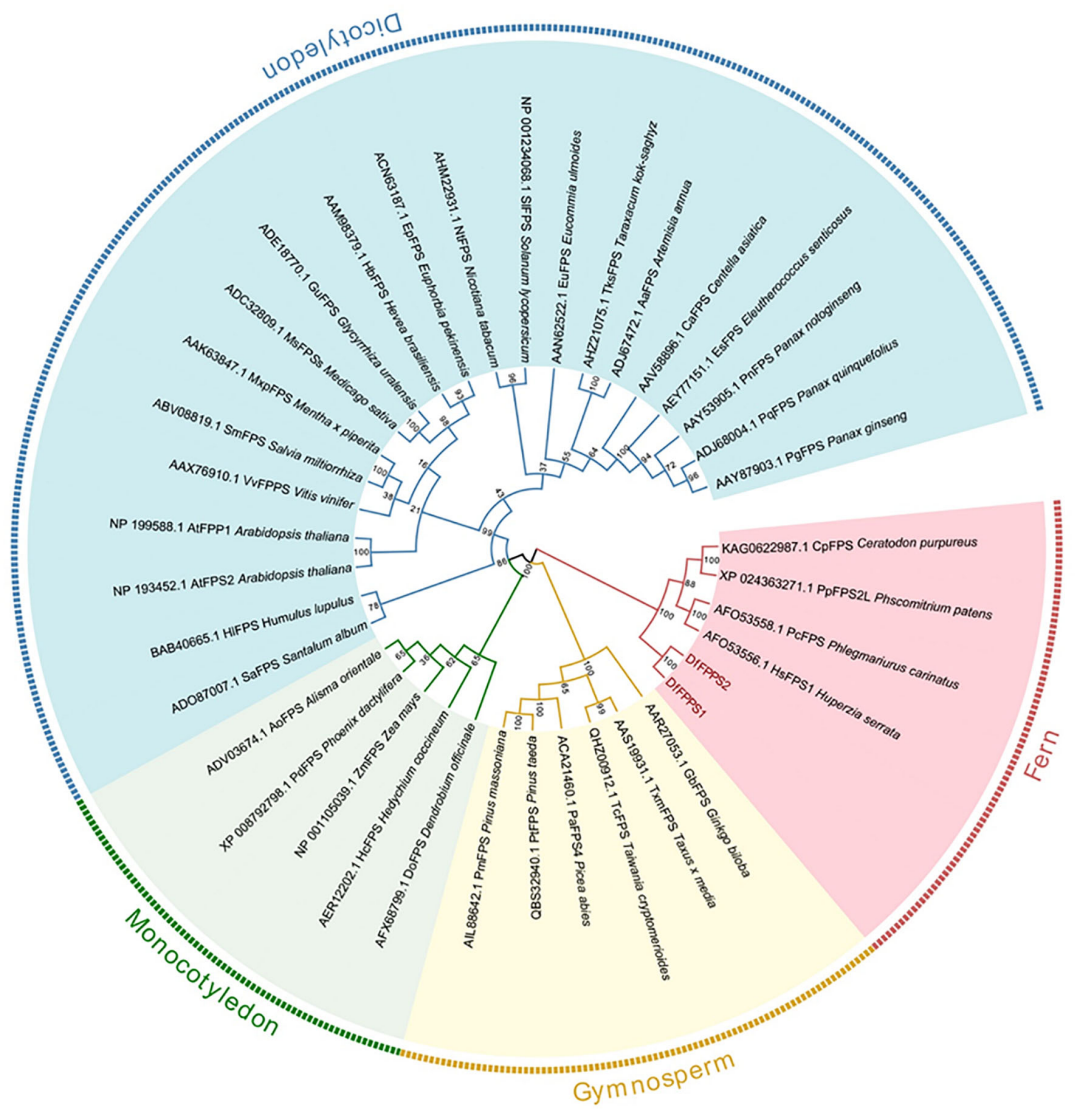
Phylogenetic tree of DfFPS1 and DfFPS2 proteins. Phylogenetic analysis of FPS from terrestrial plants; FPS protein sequences from different plants were aligned using ClustalX, and the tree was constructed using the neighbor-joining (NJ) method with MEGA 7.0.

We used NCBI BLAST to screen the *DfFPS* gene sequences from the transcriptome data of *D fragrans.* The primers designed to amplify the genes and the PCR results are shown in **Supplementary Figure S1**. Sequence analysis revealed that the sequences of the amplified genes were consistent with the transcriptome data, and the open reading frames (ORF) of *DfFPS1* and *DfFPS2* were 1041 and 1065 bp in length, respectively. The *DfFPS1* gene encoded a protein with 346 amino acids (39.87 kDa) and a predicted isoelectric point of 5.56; the *DfFPS2* gene encoded a protein with 354 amino acids (40.67 kDa) and a predicted isoelectric point of 4.94. A BLAST search of the NCBI protein database revealed that the deduced amino acid sequences of DfFPS1 and DfFPS2 were closely related to the FPS protein of other terrestrial non-seed plants, such as *Ceratopteris richardii*, *Huperzia serrata*, *Ceratodon purpureus*, and *Physcomitrium patens*. Conserved between two proteins, the nucleotide sequence identity was 84.23% and the amino acid identity was 86.72%. Next, the sequences were compared with the amino acid sequence of *A. thaliana* FPS (AtFPS), which revealed that the DfFPS proteins had the same five conserved domains as the AtFPS proteins, which are numbered I to V (Chen et al., 1994) (**Supplementary Figure S2**). The highly conserved aspartate-rich motif in region II with the amino acid sequence DDXX(XX)D is called FARM (first Asp-rich motif), which is highly conserved in all known prenyltransferases. Region V with the amino acid sequence DDXXD is called SARM (second Asp-rich motif). These regions (marked with lines) are characteristic of prenyltransferases, which can be used to synthesize isoprenoid diphosphates (Zhao et al., 2015). To explore the genetic relationship between DfFPS1 and DfFPS2 and FPS from other plant species, 36 FPS amino acid sequences of different plants were selected (**Supplementary Table S1**) and a phylogenetic evolutionary tree was constructed using MEGA6. The results revealed that the FPS proteins of dicotyledons, monocotyledons, gymnosperms, and ferns were clustered in different evolutionary clades. The two DfFPS proteins clustered in a small branch and were closely related to FPS from *Huperzia serrata*, *Phlegmariurus carinatus*, *Physcomitrium patens*, and *Ceratodon purpureus* (**Figure 1**).

### Protein tertiary structure prediction and binding site analysis

**Figure 2:**
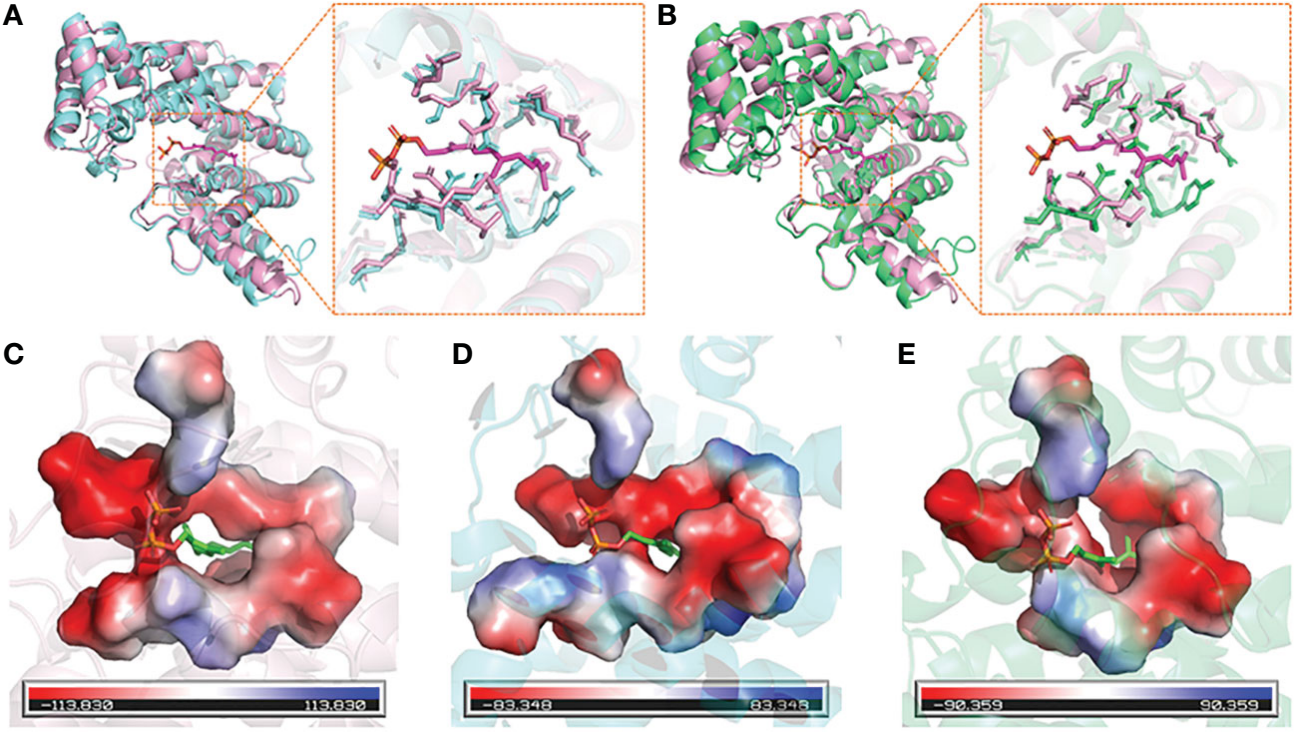
3D structures of DfFPS1 and DfFPS2 proteins. **(A)** Predicted DfFPS1 protein compared with 1ubx.1.A; **(B)** Predicted DfFPS2 protein compared with 1ubx.1.A; **(C)** FPP-binding pocket of 1ubx.1.A; **(D)** FPP binding pocket of predicted DfFPS1 protein; **(E)** FPP-binding pocket of predicted DfFPS2 protein.

The 3D structures of DfFPS1 and DfFPS2 were predicted using the online tool SWISS-MODEL, and the figures were prepared using PyMOL. All prediction models were built using ProMod Version 3.70 with x-ray at 2.20 Å. The template (SMTL ID: 4kk2.1.A) used for the 3D structure modeling of DfFPS1 had 66.37 and 50% sequence identity and similarity, respectively, with DfFPS1, and the coverage was 99%. The template (SMTL ID: 7bux.1.A) used for the 3D structure modeling of DfFPS2 had 68.13 and 51% sequence similarity and identity, respectively with DfFPS2, and the coverage was 99%. The two predicted proteins sequence were described as FPS, which were consistent with the results obtained for the cloned gene. Next, we compared the amino acid sequence of the two proteins with that of 1ubx.1.A, obtained from the SWISS-MODEL database, which is described as FPS with ligands FPP. We compared the structures of the two predicted proteins with that of 1ubx.1.A and found that the FPP binding site and amino acid residues of the two predicted proteins were the same of those of 1ubX.1.A (**Figures 2A, B** **)**. We also analyzed the binding sites of the proteins, which revealed that DfFPS1, DfFPS2, and lubx.1.A had similar binding sites for FPP (**Figures 2B–D** **)**.

### Tissue-specific and inducible expression of *DfFPS1* and *DfFPS2*

**Figure 3:**
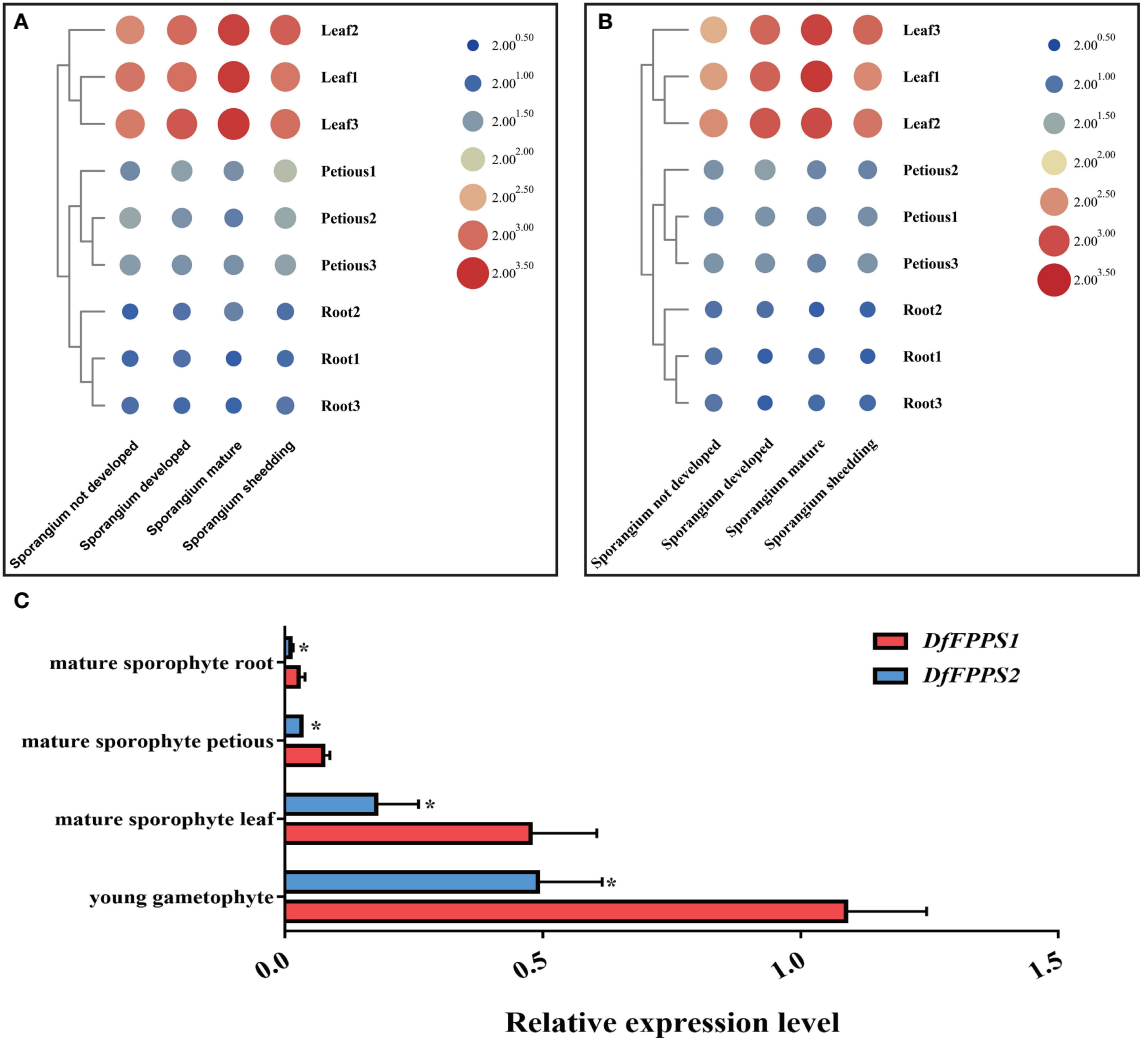
Tissue specific expression of *DfFPS1* and *DfFPS2*. **(A)** Expression pattern of *DfFPS1* in different stages and parts of *Dryopteris fragrans*; **(B)** Expression pattern of *DfFPS2* in different stages and parts of *Dryopteris fragrans*; **(C)** Comparison of expression patterns of the two *DfFPS* genes at the same site. *p < 0.05.

To explore the expression pattern of the two *DfFPS* genes, qPCR was performed to analyze their relative expression levels in different growth-stages and parts of *D. fragrans*. The analyses revealed that both *DfFPS* genes were expressed in the roots, stems, and leaves, and their expression levels in the roots and petioles in different stages varied. The expression levels of the two genes in the leaves were significantly different based on growth-stages in the following order: sporangium mature and developed > sporangium shedding > sporangium not developed (**Figures 3A, B** **)**.

However, based on the similarity in the expression patterns of the two genes in different tissues, we investigated whether this pattern is observed in other species, such as *A. thaliana*, which has two *FPS* genes that show a complementary pattern in seeds (Keim et al., 2012). *AtFPS1* is the main gene in the process of plant growth and development (Closa et al., 2010). Therefore, we speculated whether one of the two genes in *D. fragrans* was acting as the main effect gene. We performed a comparative analysis of the expression of the two genes in the leaves, petioles, and roots of mature sporophytes and young gametophytes of *D. fragrans*. We found that the expression level of *DfFPS1* was higher than that of *DfFPS2* in these tissues (**Figure 3C**).

**Figure 4:**
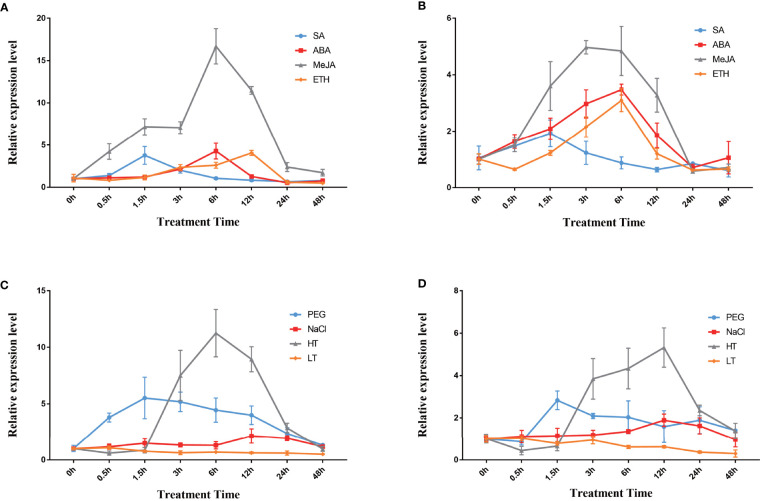
*DfFPS1* and *DfFPS2* under hormone and abiotic stress treatments. **(A)** *DfFPS1* expression level under hormones treatments; **(B)** *DfFPS2* expression level under hormone treatments; **(C)** *DfFPS1* expression level under abiotic stress treatments; **(D)** *DfFPS2* expression level under abiotic stress treatments.

Moreover, qPCR analysis was performed to examine the changes in the expression of the two *DfFPS* genes under salicylic acid (SA), ABA, MeJA, and ethylene (ETH) treatments. The expression level of *DfFPS1* first increased and then decreased under the four hormone treatments. *DfFPS1* mainly responded to MeJA treatment, and its expression began to increase at 0.5h after MeJA treatment and reached the maximum at 24h, which was 11.47 times of that observed at 0h. *DfFPS1* also responded to SA, ABA, and ETH treatments, and reached the maximum value at 1.5h, 6h, and 12h, with the expression levels being 3.77, 4.28, and 4.06 times of that at 0h, respectively (**Figure 4A**). The expression pattern of *DfFPS2* under hormone treatment was similar to that of *DfFPS1* (**Figure 4B**). However, the change in the expression level of *DfFPS2* was unobvious after treatment. Meanwhile, the gene mainly responded to MeJA treatment, reaching 3.48-folds after 6h of treatment compared with that at 0h.

Abiotic stress can promote the production of secondary metabolites in plants (Wang et al., 2021). FPS is a key enzyme of the terpenoid metabolic pathway; hence, we hypothesized that *FPS* may be induced by abiotic stress. We investigated the expression patterns of the two genes under four types of abiotic stress. We found that *DfFPS1* mainly responded to high temperature stress, followed by drought stress, but its response against salt stress and low temperature stress was negligible (**Figure 4C**). The gene expression level began to increase after 1.5h of high temperature treatment, reached 11.25 times after 6h of treatment compared with that at 0h, and returned to normal at 48h; after 1.5h of 20% PEG solution–simulated drought treatment, the expression level increased to 5.51 times of that at 0h and gradually decreased with time; with 200 mM NaCl treatment, at 12h–24h, the gene expression level increased to twice of that at 0h and decreased to original level at 48h; *DfFPS1* expression did not change significantly under low temperature treatment. The expression pattern of *DfFPS2* under stress revealed that it mainly responded to high temperature and drought stress but was not sensitive to salt stress and almost does not respond to low temperature stress (**Figure 4D**). The maximum expression of *DfFPS2* was observed after 12h of high temperature stress, which was 3.98 times of that at 0h.

### Subcellular localization of DfFPS1 and DfFPS2

**Figure 5:**
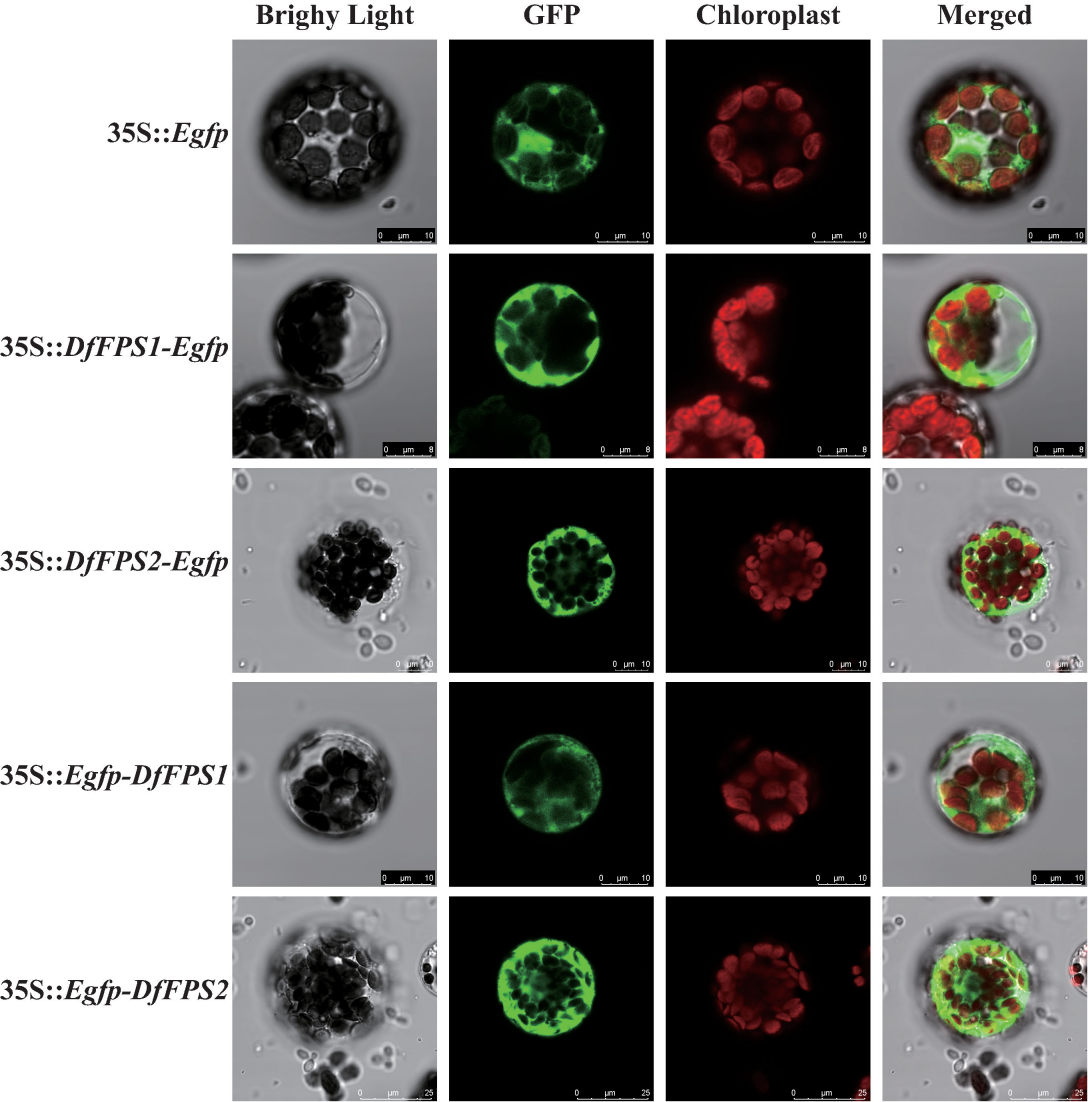
Subcellular localization of Egfp-tagged DfFPS1 and DfFPS2 proteins.

Using the PEG-mediated genetic transformation method, empty vector T-Egfp and fusion vectors T-DFFPSs-Egfp and T-Egfp-DFFPSs were transformed into *Arabidopsis* protoplasts. The protoplasts were cultured in the dark at 22–24°C for 12h–16h and observed using a confocal laser scanning microscope. The results are shown in **Figure 5**. We observed a green fluorescence signal in the nucleus, cytoplasm, and membrane of *Arabidopsis* protoplasts transformed with the empty vector T-Egfp; the cytoplasm of *Arabidopsis* protoplasts transformed with T-DFFPS1-Egfp, T-DFFPS2-Egfp, T-Egfp-DFFPS1, and T-Egfp-DFFPS2 plasmids also exhibited green fluorescence signals. These results indicated that DfFPS1 and DfFPS2 proteins were localized in the cytosol of the *Arabidopsis* protoplasmic cells.

Confocal laser scanning micrographs showing the distribution of fluorescence signals in *Arabidopsis* protoplasts.

### Prokaryotic expression and purification of DfFPS proteins

The entire reading frame of *DfFPS* genes was cloned into pET-32a(+) vector and expressed in *Escherichia coli* BL21 (DE3) cells to obtain DfFPS1 and DfFPS2 proteins for the characterization of FPS activity.

After induction by 0.5, 1.0, and 1.5 mM isopropyl thio-β-galactoside (IPTG) at 28°C and 200 rpm for 6h, the recombinant proteins were expressed at the same time, and we observed that the target protein content in the supernatants of both proteins was higher under 1.5 mM-IPTG treatment. The molecular mass of DfFPS1 (**Supplementary Figure S3A**) fused with a Trx-tag and a His-tag on N-terminal was approximately 57.6 kDa and that of the DfFPS2 (**Supplementary Figure S3B**) fusion protein was approximately 55–70 kDa, as determined by sodium dodecyl sulfate-polyacrylamide gel electrophoresis (SDS-PAGE) analysis. For all further experiments, 1.5 mM IPTG was selected for the induction of protein and the His-tagged proteins were purified using a Ni-column. And obtain purified TrxA-DfFPS1 (**Supplementary Figure S3C**) and TrxA-DfFPS2 (**Supplementary Figure S3D**) proteins.

### Catalytic activity of DfFPS proteins *in vitro*

In seed plants, FPS can condense IPP and DMAPP to form FPP under suitable conditions (Kajiura et al., 2017). To validate the function of DfFPS proteins *in vitro*, IPP and DMAPP were used as substrates to perform enzyme assays at pH 7.0 and 30°C for 30 min. The enzymatic properties of purified DfFPS1 and DfFPS2 proteins were validated by comparing the activity of the two proteins inactivated at high temperature.

**Figure 6:**
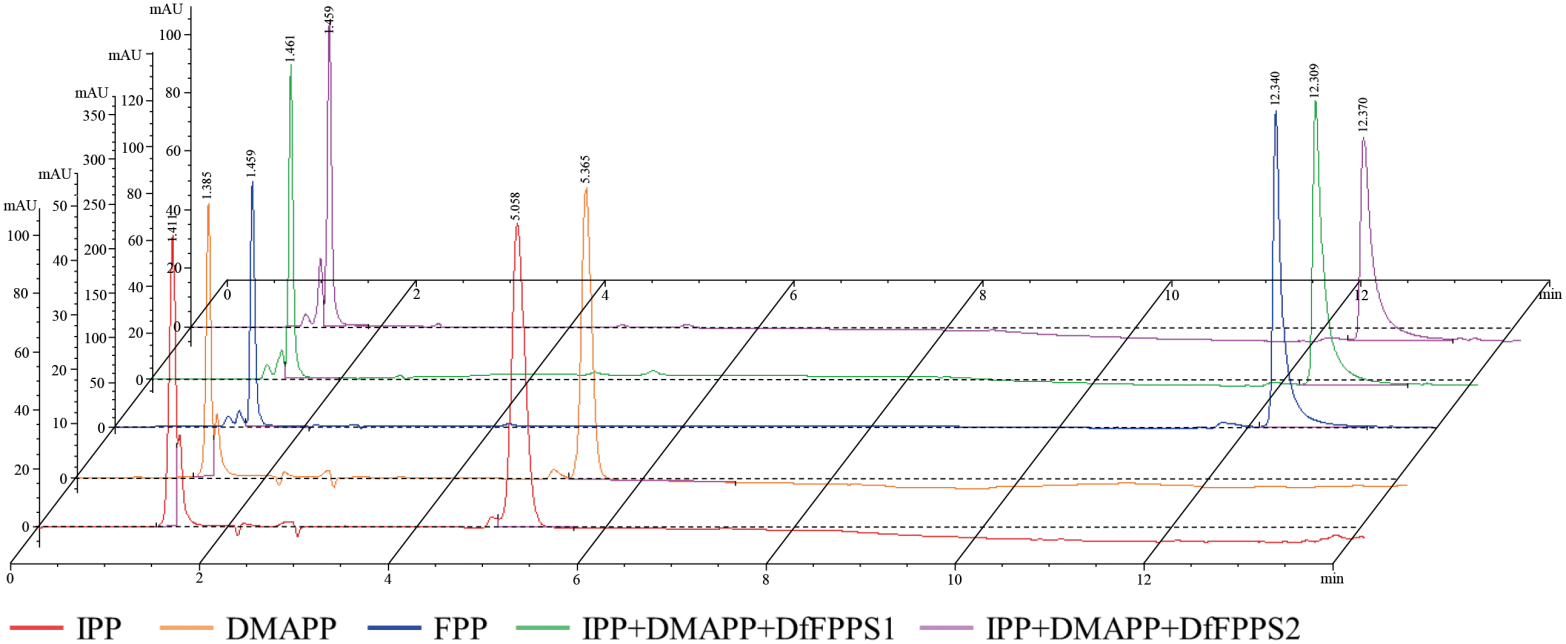
Catalytic activity of DfFPS proteins.

High-performance liquid chromatography (HPLC) analyses of the standards and enzyme-activated products are presented in **Figure 6**. In the figure, the red line is the IPP standard, which has a retention time (RT) of 5.058 min; the orange line is the DMAPP standard, which has an RT of 5.365 min; the blue line is the FPP standard, which has an RT of 12.340 min; the green line is the reaction between IPP and DMAPP as substrates at pH 7.0 and 30°C for DfFPS1 protein, and the RT of the product is 12.309 min, same with FPP standard; the purple line is the reaction between IPP and DMAPP as substrate at pH 7.5 and 30°C for DfFPS2 protein, and the RT of the product is 12.370 min, same with FPP standard.

### Phenotypic analysis of *DfFPS* genes in transgenic tobacco plants

Eight and seven transgenic tobacco plants overexpressing *DfFPS1* and *DfFPS2* were obtained, respectively, by agrobacterium-mediated genetic transformation (**Supplementary Figure S4**).

**Figure 7:**
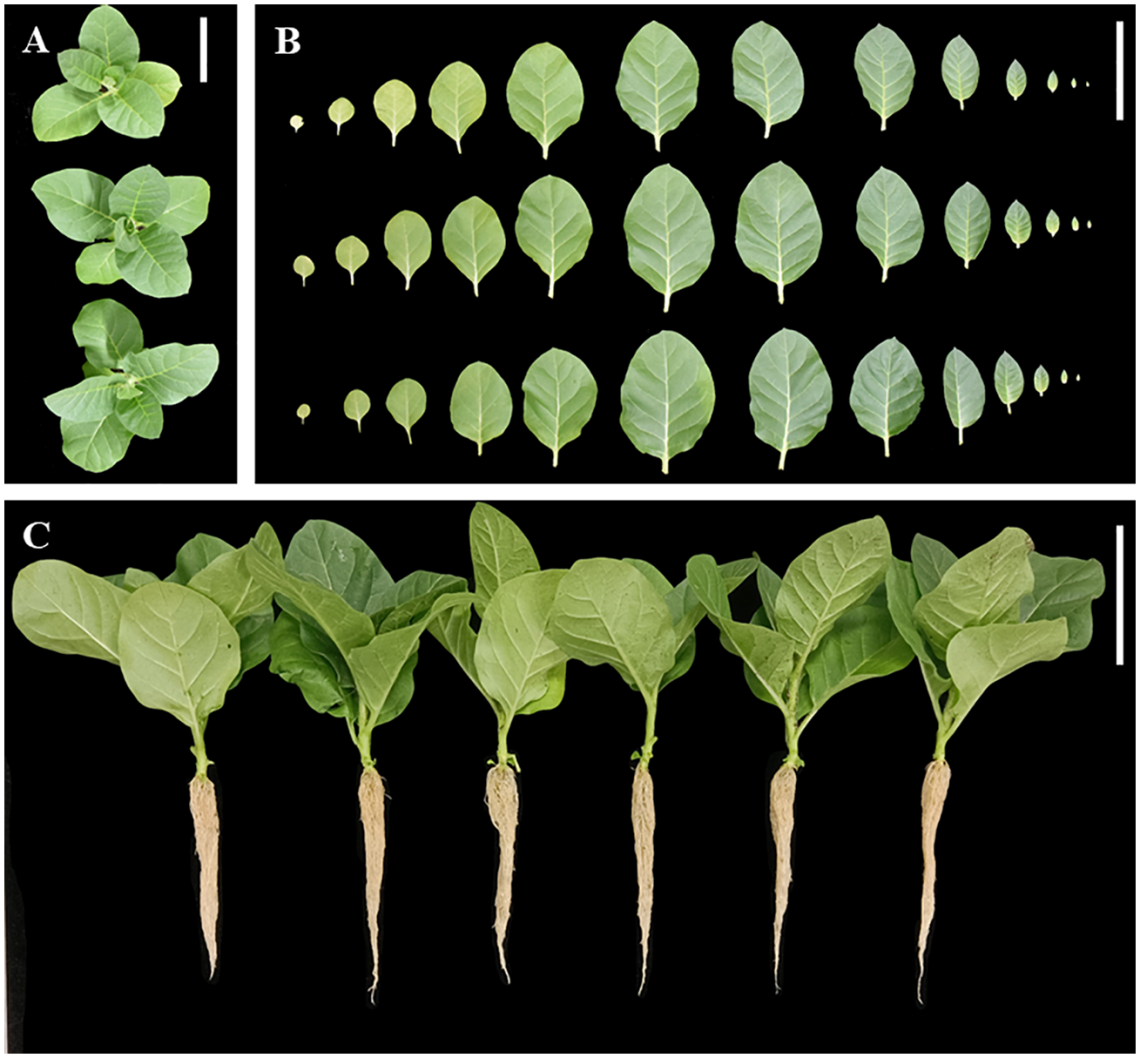
Growth and development analyses of transgenic and wild-type tobacco plants. **(A)** Top view of tobacco plants; top to bottom: wild type, *35S::FPS1-8*, and *35S::FPS2-4*; **(B)** Tobacco leaves (in the same order as described in **Figure 7A**); leaf blade from left to right was 1^st^ to 13^th^ of tobacco plants; **(C)** normal development of tobacco plants; left to right: wild-type, vector control, *35S::FPS1-8*, *35S::FPS1-9*, *35S::FPS2-4*, and *35S::FPS2-6.* (Bars = 10 cm).

**Figure 8:**
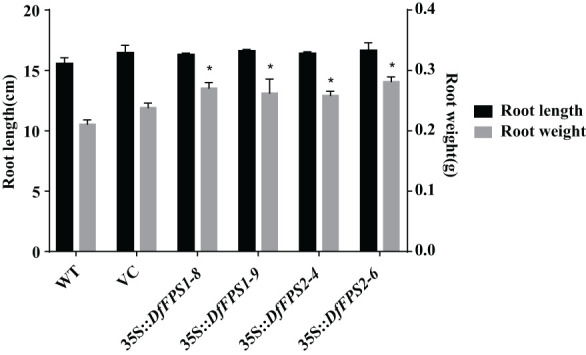
Length and dry weight of roots of transgenic and wild-type tobacco plants. *p < 0.05.

Wild-type seedlings and T2 transgenic tobacco seedlings were grown up to the four-leaf stage in the medium, transferred to nutrient soil for culture, and the growth and development of the plants were observed after 21 days. Wild-type, *35S::DFFPS1-8*, and *35S::DFFPS2-4* tobacco plants are presented in **Figures 7A, B** (from top to bottom). A significant difference was not observed between transgenic and wild-type tobacco plants. The length and dry weight of transgenic plants was measured (**Figures 7C**, **8**), which revealed that the root length of transgenic plants was not significantly different from that of wild-type and empty vector plants; however, the root dry weight of transgenic plants increased significantly compared with that of wild-type and empty vector plants, whereas a significant difference was not observed between the root dry weight of *35S::DfFPS1-8/9* and *35S::DfFPS2-4/6* transgenic tobacco plants.

**Figure 9:**
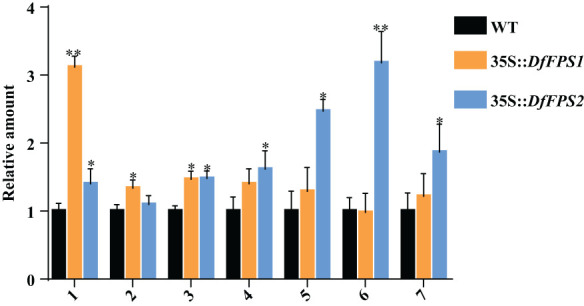
Amount of metabolites in wild-type and *DfFPS*-overexpressing tobacco plants. 1, nicotine; 2, neophytadiene; 3, n-docosanol; 4, cis-7,10,13,16,19-docosapentaenoic acid methyl ester; 5, manool; 6, geranyl linalool; 7, eicosapentaenoic acid ethyl ester. *p < 0.05; **p < 0.01.

FPS is the key enzyme of the terpene metabolic pathway. Overexpression of *FPS* genes may affect the expression of terpene metabolites in plants; therefore, we performed gas chromatography-mass spectrometry (GC-MS) to detect the metabolites in tobacco leaves. The leaves of the same part and growth stage were selected from the transgenic tobacco lines and were mixed in equal mass. GC-MS analyses revealed that the RT of the standard nonyl acetate was 7.433 min. Moreover, the main volatile secondary metabolites in tobacco were (1) nicotine, (2) neophytadiene, (3) n-docosanol, (4) cis-7,10,13,16,19-docosapentaenoic acid methyl ester, (5) manool, (6) geranyl linalool, and (7) eicosapentaenoic acid ethyl ester (**Supplementary Figure S5**). Qualitative and quantitative analysis of tobacco leaves using nonyl acetate revealed that the nicotine content of *35S::DfFPS1* transgenic tobacco leaves was 3.12 times that of the wild-type leaves and 1.47 times that of neophytadiene content; cis-7,10,13,16,19-docosapentaenoic acid methyl ester, manool, geranyl linalool, and eicosapentaenoic acid ethyl ester contents in *35S::DfFPS2* transgenic tobacco leaves were 1.62, 2.47, 3.18, and 1.87 times those of wild-type tobacco leaves, respectively (**Figure 9**).

**Figure 10:**
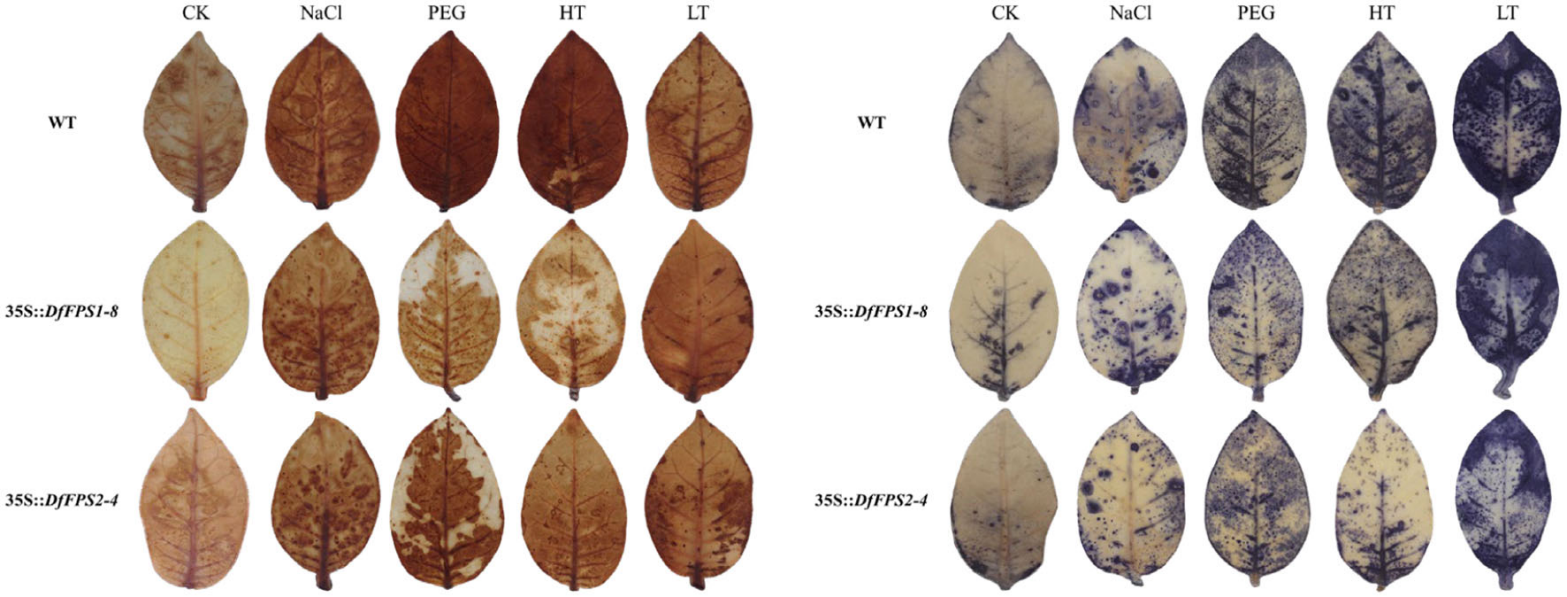
NBT and DAB staining of wild-type and transgenic tobacco leaves (*35S::DfFPS1-8* and *35S::DfFPS2-4*) under different types of abiotic stress.

Based on the fact that the *DfFPS* genes were induced by abiotic stress, we speculated that the overexpression of these two genes might lead to a stress-resistant phenotype in tobacco. Four-week-old transgenic and wild-type tobacco plants were selected and subjected to 150 mM NaCl treatment (salt stress), 20% PEG (drought stress), 42°C (high temperature stress), or 4°C (low temperature stress). Next, the leaves from the same parts of the plants were selected for nitro blue tetrazolium (NBT) and 3, 3’-diaminobenzidine (DAB) staining to preliminarily verify gene function. The leaves of *DfFPS1*- and *DfFPS2*-overexpressing tobacco plants under high temperature and drought stress were lighter than those of the wild-type plants, whereas the leaves of transgenic and wild-type tobacco plants were similar in color under low temperature and salt stress. NBT and DAB staining revealed similar patterns for these treatments (**Figure 10**, **S6**). The results revealed that transgenic tobacco plants exhibited a certain degree of resistance to high temperature and drought stress.

### Transcriptional activity analysis of *DfFPS* gene promoters

In the present study, *DfFPS* genes were found to respond to MeJA treatment and drought and high temperature stresses, and the transgenic plants exhibited a certain degree of resistance to high temperature and drought. However, the underlying mechanisms responsible for the stress response exhibited by the transgenic plants are unclear; therefore, we cloned the promoter sequences of the two *DfFPS* genes to analyze their transcriptional activity.

Since the genome of *D. fragrans* is not sequenced yet, we used fusion primer and nested integrated PCR (FPNI-PCR) for genome walking to amplify the promoter sequence of the gene from the predicted coding sequence (CDS) (Wang et al., 2011). The genome walking results of *DfFPS1* revealed that none of the FP primers could successfully amplify the gene (**Supplementary Figure S7A**). The genome walking results of *DfFPS2* revealed that the FP2/3/4/5/7 primers (of the nine random primers) could successfully amplify the promoter (**Supplementary Figure S7B**). Sequencing analyses revealed that the amplicon obtained using the FP2 primer could coincide with the sequence of the 5’-UTR of *DfFPS2*, and the length of the promoter of *DfFPS2* was 1554 bp. Since the promoter sequence *DfFPS1* could not be amplified using the FP1-9 primers, seven new FP primers (FP10-16) were designed according to the primer design principle described in the materials and methods section. FPNI-PCR using the FP11/14/15 primers successfully amplified the *DfFPS1* promoter (**Supplementary Figure S7C**). Sequencing analysis revealed that three sequences were the same and could be compared with the 5’-UTR sequence of *DfFPS1*. Since the amplicon obtained using the FP15 primer was long (943 bp), this sequence was selected for subsequent experiments.

**Figure 11:**
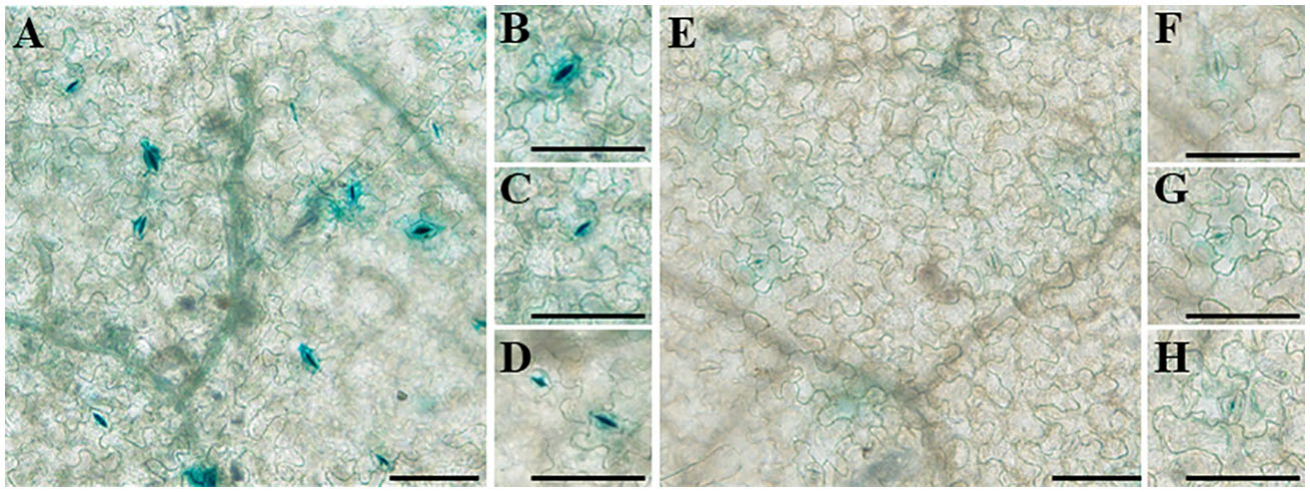
Transcriptional activity of full-length *DfFPS* promoters in tobacco leaves. **(A–D)** *DfFPS1* promoter localization; **(E–H)** *DfFPS2* promoter localization. (Bars = 100 μm).

To analyze the transcriptional activity of the full-length promoters of the two genes, the promoter sequences of the two genes were cloned into pBI121 upstream of the β-glucuronidase (GUS) reporter. *GUS* expression driven by the two promoters was high in the stomata of tobacco leaves, which has not been reported earlier (**Figure 11**). Moreover, the transcriptional activity of proDfFPS1 was higher than that of proDfFPS2, which is similar to qPCR results (**Figure 3C**).

**Figure 12:**
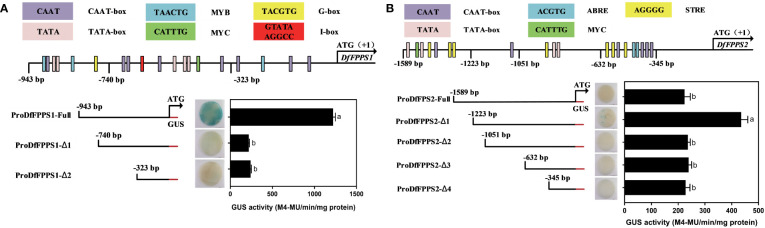
Transient element prediction and transcriptional activity analysis of *DfFPS* promoters truncated to different lengths. **(A)** Analysis of truncated *DfFPS1* promoter and its transcriptional activity; **(B)** Analysis of truncated *DfFPS2* promoter and its transcriptional activity.

To determine the active site of the promoters, according to the PlantCare-based predictions, *DfFPS1* and *DfFPS2* promoters were truncated into different lengths according to cis elements to control transient expression of *GUS* in tobacco leaves. *proFPS1-Full* exhibited high transcriptional activity, whereas *proFPS1-Δ1* and *proFPS1-Δ2* exhibited negligible transcriptional activity, indicating that the sequence between *proFPS1-Full* and *proFPS1-Δ1* could be the transcriptionally active region of *DfFPS1* (**Figure 12A**). We also found that the transcriptional activity of *DfFPS2* promoter with different lengths was low, with almost no transcriptional activity (**Figure 12B**).

**Figure 13:**
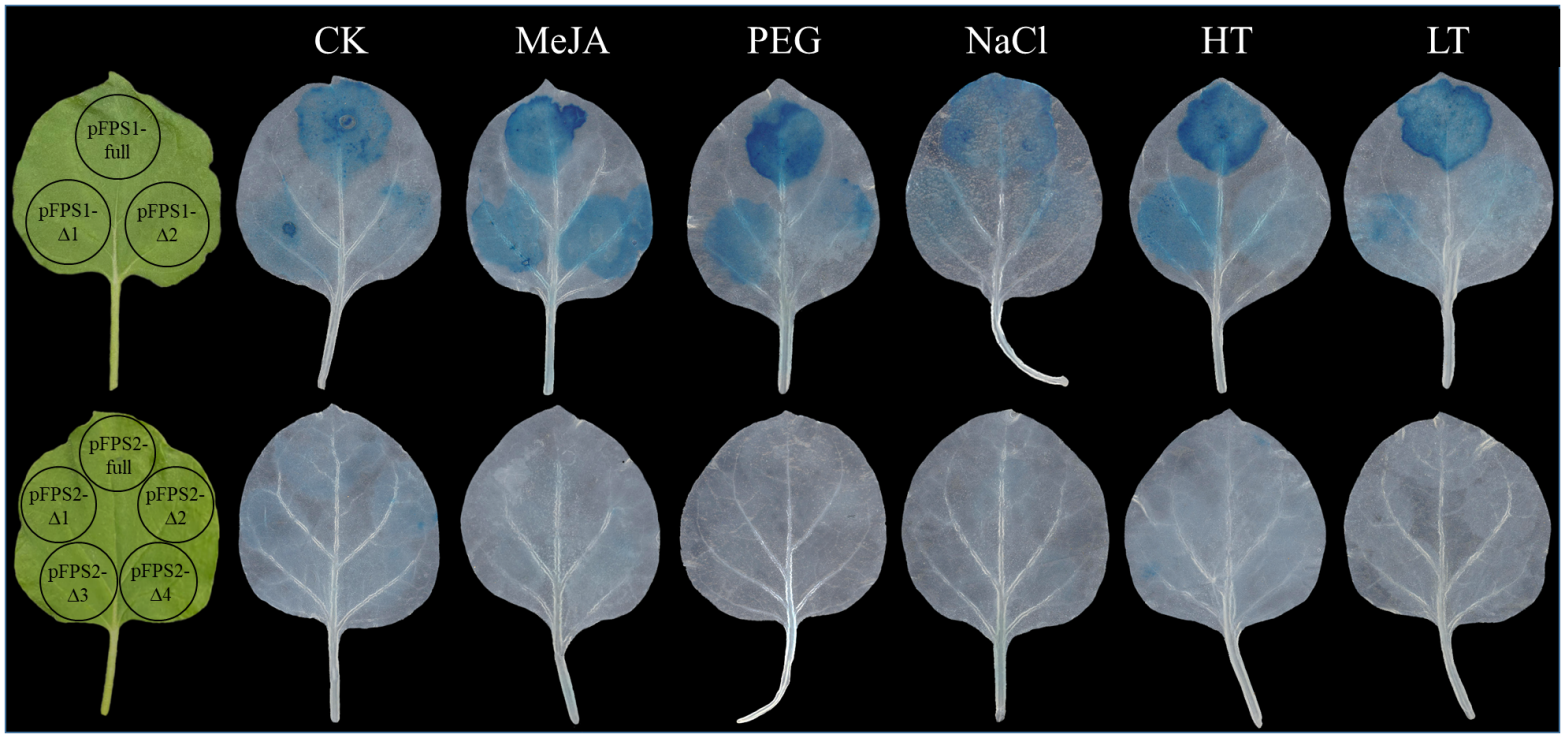
Transcriptional activity of truncated *DfFPS* promoters under different treatments.

Next, we analyzed the response of the promoters of different lengths to stress, and we selected the leaves of the same parts of each plant for transformation. To ensure the accuracy of the experiment, different truncated fragments of the same promoter were injected into the same leaf, and the plants were treated with MeJA, 150 mM NaCl, 20% PEG, 42°C, or 4°C for 6h after 3 days of transformation. The results revealed that the transcriptional activity of the truncated *DfFPS1* promoter was changed to some extent under MeJA treatment and abiotic stress, and the main transcriptional active site of *DfFPS1* promoter was the fragment between *proFPS1-Full* and *proFPS1-Δ1*. Similarly, under different treatments, the transcriptional activity of *DfFPS2* promoters of different lengths under MeJA and abiotic stress was low (**Figure 13**).

## Materials and methods

### Plant material and treatments

*D. fragrans* spores were collected from lava rocks near Bagua Lake, Wudalianchi, Heilongjiang, China (48.733334 N, 126.167966E) within a 30-m radius during July 1–6, 2018, after obtaining necessary permission from the government. To ensure uniformity in samples, we used *D. fragrans* that were grown at 25°C under a 16h/8h photoperiod for 2 years.

The leaves, petioles, and roots of *D. fragrans* were selected from 2-year-old plants at different stages, that is, undeveloped, developed, mature, and shedding sporangia. Two-year-old *D. fragrans* sporophytes were subjected to the following hormone treatments: 100 μM SA, 10 μM ABA, 120 μM MEJA, and 500 μM ETH, and water was used as the control; they were also subjected to the following four types of abiotic stress: 200 mM NaCl (salt stress), 20% PEG irrigation (drought stress), 42°C (high temperature stress), 4°C (low- temperature stress), and 24°C and water irrigation (control). The samples were collected, immediately frozen in liquid nitrogen, and stored at −80°C until further use.

*A. thaliana* ecotype Columbia-0 (Col-0) was grown in a growth chamber under a photoperiod of 16 h/8h (light/dark) at 22°C.

*Nicotiana tabacum* L. (Shan Xi) seeds were sterilized and cultured in 1/8 MS medium (M519; Phytotech ,Lenexa, USA). After 4 weeks, the leaves were cut into 1-cm square pieces and placed on M1 medium (MS agar medium containing 0.1 mg/liter NAA and 1 mg/liter 6-BA) for 2–3 days. The prepared agrobacterium culture was adjusted to an OD_600_ of 0.8 using sterile water and transferred to the leaves for 8–10 min; next, the culture was drained and returned to the original medium. After 4 days of culture, the leaves were washed with sterile water and transferred to M2 medium (M1 medium containing 50 mg/liter Kan and 50–400 mg/liter Cef) until resistant buds developed. The resistant buds were grown up to a height of 2 cm and then transferred to 1/2 MS medium for rooting.

Four-week-old *N. tabacum* L. plants were used to investigate instantaneous expression, and the plants were watered a day before transformation. The prepared agrobacterium culture was adjusted to an OD_600_ of 0.6 by injecting a buffer (10 mM MgCl_2_, 10 mM MES, and 150–200 μM AS). The bacterial solution (1 ml) was injected using a medical syringe into the tobacco leaves from the lower epidermis through the stomata. After injection, the plants were kept at 22–24°C for 1 day in dark and then in low light for 1 day, followed by growth under normal conditions for 2 days.

Wild-type and T2 transgenic tobacco seedlings were grown to the four-leaf stage in the MS medium, then transferred to nutrient soil for further growth, and their growth and development were observed after 21 days. Simultaneously, the plants were subjected to the following four types of abiotic stress: 200 mM NaCl (salt stress), 20% PEG irrigation (drought stress), 42°C (high temperature stress), 4°C (low-temperature stress) for 12h; then, the leaves of the same part were stained using NBT and DAB.

Transient transformed tobacco plants were subjected to 120 μM MeJA treatment and the abovementioned abiotic 12h before sampling, and the leaves were stained using X-gluc solution.

### Cloning of *DfFPS* genes

RNA of *D. fragrans* was extracted from the sporophyte leaves using a Trelief TM RNAprep Pure Plant Plus Kit (Polysaccharides & Polyphenolics-rich; Tsingke, China), and reverse transcription was performed using a HiScript III 1st Strand cDNA Synthesis Kit (+gDNA wiper) (Vazyme, China) to obtain cDNA. The putative *DfFPS* genes were amplified using 2×Phanta^®^ Max Master Mix (Vazyme, Nanjing, China) with the following primer sets: DfFPS1 (F: 5′-ATGTCCAAGGATGCATCAGG-3′; R: 5′-TCATTTTTGTCTTTTGTAAATTTTTCCCAAG-3′); DfFPS2 (F: 5′ATGGCTCCTACTGTGGAACT-3′; R: 5′-TCATTTTTGTCGTTTGTAGATTTTTCCTAAG-3′). The obtained amplicons were inserted in a vector (pClone007 Versatile Simple Vector Kit), and the plasmid was sequenced.

### Sequence analysis

The nucleotide and protein sequences were compared using the NCBI tools (http://www.ncbi.NLM.NIH.gov). The ORF of the two genes was identified using ORF Finder (www.ncbi.NLM.NIH.gov/Gorf/Gorf.html). Multiple sequence alignments were performed using the DNAMAN software. Phylogenetic analysis was performed using the MEGA 6.0 software, and the sequences were aligned using ClustalW. A phylogenetic tree was constructed using Evolview (https://www.evolgenius.info/evolview/#login) and was based on the P-distance model of the neighbor-joining method, with partial deletion and interior-branch test replication being 1,000 times (Tamura et al., 2013) to construct the evolutionary tree. The 3D structures of DfFPS1 and DfFPS2 were predicted using the SWISS-MODEL online tool (https://swissmodel.expasy.org/) (Bienert et al., 2017), and figures were prepared using PyMOL.

### qPCR for *DfFPS1* and *DfFPS2* expression analysis

RNA was extracted as described above; for cDNA synthesis, 1000 ng of total RNA was mixed with the HiScript^®^ II Q RT SuperMix for qPCR (+gDNA wiper) (Vazyme, China). We performed qPCR using ChamQ Universal SYBR qPCR Master Mix (Vazyme, China), as per the manufacturer’s instructions. The primers were designed using Primer Premier 6 (**Supplementary Table S2**). The transcript levels were normalized to that of the 18*S* rRNA gene of *D. fragrans*. The relative expression levels were calculated using a previously reported formula (Livak and Schmittgen, 2001). All biological replicates were measured in triplicate, and a plot was constructed using GraphPad Prism 7 and TBtools.

### Protoplast transient transfection assay

To verify the subcellular localization of DfFPS proteins, fusion plasmids T-DFFPS1-Egfp, T-DFFPS2-Egfp, T-Egfp-DFFPS1, and T-Egfp-DFFPS2 were constructed using the Gibson assembly. All plasmids used in the transient transfection assays were endotoxin free. The plasmids were extracted using the GoldHi EndoFree Plasmid Maxi Kit (CWBIO), and their concentration was adjusted before transfection to approximately 4000 ng·µl^−1^ using isopropanol and 5 M NaCl solution. The transient *Arabidopsis* protoplast transfection assays were performed as previously described (Yoo et al., 2007). Briefly, 3- to 4-week-old rosette leaves of wild-type *Arabidopsis* plants were harvested and immediately cut into strips using a razor. The strips were incubated in an enzyme solution containing 1% (w/v) cellulase R10 (Yakult Pharmaceutical Ind. Co., Ltd, Japan) and 0.25% (w/v) macerozyme R10 (Yakult Pharmaceutical) at 22–24°C for 3h. The protoplasts were purified, and the fusion plasmids were transfected into the protoplasts using PEG. After transformation, the protoplasts were cultured at 22–24°C for 12–16h under dark conditions, and they were observed using a confocal laser microscope.

### Expression of *DfFPS1* and *DfFPS2* in *E. coli* and purification of recombinant protein

To verify the enzyme activity of the two proteins, the genes encoding the proteins were cloned in a prokaryotic expression vector [pET32a(+)-DfFPS1 and pET32a(+)-DfFPS2] using the Gibson assembly. *E. coli* BL21 (DE3) cells were used for recombinant protein expression; the protein was induced using 1 mM IPTG at 16°C for 20h. After the cells were harvested, they were resuspended in 2 ml of lysis buffer A [20 mM tris-HCl (pH 8.0), 500 mM NaCl, 1 mM PMSF, and bacterial protease inhibitor cocktail], lysed by sonication (sonication for 5 s, followed by pause for 5 s), centrifuged for 30 min at 13,000 × *g*, and the protein was harvested from the supernatant.

The supernatant was loaded on a Ni-NTA column, which was pre-equilibrated using lysis buffer A. The column was washed with 10 column volumes of lysis buffer A containing 20 mM imidazole. The target proteins were eluted using buffer B [20 mM tris-HCl (pH 8.0) containing 500 mM NaCl and 50 mM imidazole] and buffer C [20 mM tris-HCl (pH 8.0) containing 150 mM NaCl and 250 mM imidazole). The purified proteins were subjected to SDS-PAGE. The concentration of the proteins was determined using the Bradford method.

### Enzyme activity analysis

Enzyme activity analysis and HPLC were performed as previously described (Ferriols et al., 2015), with minor modifications. The enzyme assays were performed in 1.5-ml Eppendorf tubes in a total volume of 100 μl containing 35 mM tris-HCl (pH 7.5), 10 mM MgCl2, 4 mM dithiothreitol (DTT), 50 μM isopentenyl pyrophosphate (IPP; Echelon Biosciences, Inc) and 150 μM dimethylallyl pyrophosphate (DMAPP; Echelon Biosciences, Inc) as the allylic substrates, and 100 ng of the purified enzyme. The assay mixtures were prepared in bulk without the substrates, and the reactions were initiated by substrate addition after sufficient equilibration of the system temperature. The reactions were performed at 30°C for 10 min and stopped by snap freezing the assay tubes in liquid nitrogen. To denature and precipitate the proteins before HPLC analysis, 100 μl of acetonitrile was added to the reaction mixture, and the mixture was vortexed for 10 s and centrifuged at 19,000 × *g* for 5 min at 4°C.

HPLC was performed using an Agilent Technologies 1200 Series HPLC system. The separation of the reaction product FPP was achieved on an Agilent ZORBAX SB-C18 column (particle size: 5 μm, 4.6 mm × 150 mm) using a mobile phase consisting of 5 mM NH_4_HCO_3_ and 100% acetonitrile as solvents A and B, respectively. A gradient system was used for the analyses, wherein 2% solvent B was increased to 5% in 5 min, followed by an increase of solvent B to 50% in 10 min and to 100% in 5 min, and this was maintained for 5 min. The flow rate was 0.8 ml·min^−1^; the column temperature was 30°C; the injection volume was 10 μl; and the detection wavelength was 214 nm for all samples and standards.

### Determination of the secondary metabolite content of *DfFPS* transgenic tobacco plants

Secondary metabolites from transgenic plants were detected as per a previously described method (Song et al., 2022). Briefly, we added 0.1 g of tobacco leaves (three plants of each transgenic line were selected at the four-leaf stage; the samples from both lines were mixed for the experiment) and 1 ml of ethyl acetate standard solution to a 1.5-ml Eppendorf tube, which contained 8.64 µg·ml^−1^ nonyl acetate as the internal standard. The Eppendorf tube was centrifuged using an Ultra Centrifugal Mill (700 rpm, 5 min) and ultrasonicated using a small ultrasonic cleaner (60 kHz, 40°C, 30 min). After sonication, the tube was centrifuged at low temperature (5000 × *g*, 5 min), and the supernatant was aspirated and filtered through a 0.22-µm microporous membrane for GC-MS.

Terpenoids were analyzed using an Agilent 7890A gas chromatograph coupled to an Agilent 5975C Network Mass Selective Detector (MS, insert XL MSD with triple-axis detector) and an HP-5MS column (30 m × 0.25 mm × 0.25 µm; J&W Scientific, Folsom, CA, USA). The GC-MS conditions were as follows: Carrier gas: He (purity ≥ 99.999%); injection port temperature: 280°C; injection method: splitless injection; injection volume: 1 µl. The temperature conditions were as follows: the initial temperature was 60°C for 2 min, then increased to 220°C for 1 min at 20°C·min^−1^, followed by an increase to 250°C for 1 min at 5°C·min^−1^, and final increase to 290°C for 7.5 min at 20°C·min^−1^. The MS conditions for the analyses were as follows: ion source: EI; ion source temperature: 230°C; ion energy mode: tuning setting; ion energy (eV): 70; detector setting: gain factor; solvent delay: 5 min; mass scan range: 30–500 m/z.

### FPNI-PCR-based cloning of the promoter sequences of *DfFPS* genes

The promoter was cloned using FPNI-PCR (Wang et al., 2011), and 2×Rapid Taq Master Mix (Vazyme, China) were used to obtain the promoter.

The 1^st^ PCR was a thermal asymmetric interlaced (TAIL)-PCR performed using *D. fragrans* genomic DNA as the template with FP1-9 primers (**Supplementary Table S3**) and gene specific primers DfFPS1-SP1and DfFPS2-SP1 (**Supplementary Table S4**). The amplicons from the 1^st^ PCR were used as the template for the 2^nd^ PCR, and nested PCR-specific primers FSP1 (**Supplementary Table S3**) and secondary gene specific primers DfFPS1-SP2 and DfFPS2-SP2 (**Supplementary Table S4**) were used. In the 3^rd^ PCR, the amplicons from the 2^nd^ round were diluted 100 times and used as the template, and specific primer FSP2 (**Supplementary Table S3**) and gene specific primers DfFPS1-SP3 and DfFPSP2-SP3 (**Supplementary Table S4**) were used for common PCR. The specific programs that were used are listed in **Supplementary Table S5**.

Since the promoter of *DfPS1* could not be amplified using the FP1-9 primers, we designed seven new FP primers. The sequence of primers FP10-16 is presented in **Table 1**. The same PCR procedure and gene-specific primers were used to amplify the promoter of *DfFPS1*.

**Table 1:** Sequence of the FP primers designed in this study.

Primer name	Primer sequence	Purpose
FP10	5′-GTAATACGACTCACTATAGGGCACGCGTGGT TGWGNAGSANCASAGA-3′	1^st^ PCR
FP11	5′-GTAATACGACTCACTATAGGGCACGCGTGGT STTGNTASTNCTNTGC-3′	1^st^ PCR
FP12	5′-GTAATACGACTCACTATAGGGCACGCGTGGT TGAGNAGTANCAGAGA-3′	1^st^ PCR
FP13	5′-GTAATACGACTCACTATAGGGCACGCGTGGT AGTGNAGAANCAAAGG-3′	1^st^ PCR
FP14	5′-GTAATACGACTCACTATAGGGCACGCGTGGT CATCGNCNGANACGAA-3′	1^st^ PCR
FP15	5′-GTAATACGACTCACTATAGGGCACGCGTGGT TCGTNCGNACNTAGGA-3′	1^st^ PCR
FP16	5′-GTAATACGACTCACTATAGGGCACGCGTGGT CAWCGTCNGATASGGA-3′	1^st^ PCR

### Transcriptional activity analysis of promoters

We used the plant cis-acting regulatory element (plantCARE) website (http://bioinformatics.psb.ugent.be/webtools/plantcare/html/) to predict potential cis promoter regions of the two genes (Lescot et al., 2002). The transcriptional activity of the full-length promoters of the two genes was verified by investigating the transient expression of *DfFPS* genes in tobacco leaves. The promoters for both genes were truncated according to the homeopathic element cis on the promoters, and truncated *DfFPS1* (938, 740, and 327 bp) and *DfFPS2* promoters (1554, 1223, 1051, 632, and 345 bp) with different lengths were obtained, using the primers listed in **Supplementary Table S6**.

## Discussion

The *FPS* gene, reported from several plants, participates in the secondary metabolism of plants and plays an important role in plant growth and development; therefore, it is evolutionarily conserved in several terrestrial plant species. In the present study, a phylogenetic tree was constructed, which revealed that the *DfFPS* genes and *FPS* genes from other ferns were clustered into a large clade. *In vitro* protein localization and functional analyses revealed that the DfFPS proteins were mainly localized in the cytoplasm, which was in accordance with the results reported by a previous study for AtFPS1S and AtFPS2 (Keim et al., 2012). Moreover, DfFPS could catalyze the production of FPP *in vitro*, suggesting that its function is the same as the FPS protein in other plant species. The existence of the conserved motifs ensured that the protein possesses catalytic activity.

We observed that the *DfFPS* genes were expressed in different parts of the plants during different growth stages. The expression level of the genes in the gametophyte stage was significantly higher than that in the sporophyte stage, which might play an important role in plant growth and development. In mature leaves (sporophyte stage), the expression of *DfFPS* genes was higher and secondary metabolism was more vigorous, providing substrates for development. The two *AtFPS* genes of *Arabidopsis* exhibit complementary expression patterns during seed germination (Keim et al., 2012); therefore, we investigated the expression pattern of the two *DfFPS* genes in different parts of the plants. We found that the expression level of *DfFPS1* was significantly higher than that of *DfFPS2* in the sporophyte stage and in different parts of mature gametophytes. Moreover, we elucidated that the transcriptional activity of the *DfFPS1* promoter was significantly higher than that of the *DfFPS2* promoter. Therefore, we speculated that *DfFPS1* plays an important role in the growth and development of *D. fragrans*.

Under different hormone treatments, we found that both *DfFPS* genes mainly responded to MeJA treatment. *FPS* genes from other plants also respond to MeJA. The expression of the *DfFPS* genes increased in response to drought and high temperature. However, moderate drought or high temperature has also been reported to increase secondary metabolite expression in plants (Taghinasab and Jabaji, 2020; Wen et al., 2022). Therefore, we hypothesized that drought and high temperature may increase secondary metabolite expression by promoting the expression of precursor synthase FPS.

Previous studies on the function of FPS in eukaryotic have focused on its effect on secondary metabolites and growth in plants. In this study, we used GC-MS to investigate the changes in the contents of volatile secondary metabolites in tobacco plants (leaves) by overexpressing the two *DfFPS* genes and found that of the content of several terpenoids in tobacco had increased. However, the overexpression of these genes did not exhibit any obvious changes in plant growth and development.

Moreover, we elucidated that both *DfFPS* genes responded to abiotic stress, and their expression significantly increased after PEG treatment. Therefore, we speculated that the DfFPS proteins contributed in making the plants resistant to abiotic stress to some extent. To the best of our knowledge, no studies have reported the role of *FPS* genes in the resistance exhibited by plants against abiotic stress. Here, we conducted a preliminary investigation of the resistance phenotype of tobacco plants using NBT and DAB staining and found that the transgenic plants exhibited resistance to high temperature and drought. We hypothesized that the overexpression of the *DfFPS* genes resulted in the accumulation of the sesquiterpenoid precursor FPP in the cells to ensure sufficient substrate concentration for the rapid synthesis of ABA under drought and high temperature conditions, enabling a rapid response against stress. The promoters of the two *DfFPS* genes were highly expressed in the guard cells of the stomata. Therefore, these genes may be highly expressed in the stomata to accumulate FPP for ABA synthesis, and the accumulated ABA can lead to the rapid closure of stomata, thus conferring resistance traits to the plants. However, a direct pathway for ABA production with FPP as the precursor has not been identified in plants. The results of the present study will provide a basis for future studies on the investigation of a direct pathway for ABA production.

Moreover, we optimized the FPNI-PCR process to amplify the promoter sequences of the two genes. We also performed TAIL-PCR for cloning the promoter sequences, but the accuracy of the results obtained using FPNI-PCR was higher than those obtained using TAIL-PCR. We used nine FP primers to amplify the promoter sequences, but only the promoter of *DfFPS2* could be successfully amplified. Therefore, we confirmed that the optimized FPNI-PCR method can be used for amplifying promoters and speculated that the random primer part of the FP primers may not be able to bind to the *DfFPS1* promoter. Next, we designed seven new FP primers according to the primer designing methods for TAIL-PCR and FPNI-PCR, and we amplified the promoter of *DfFPS1* using these primers; the results suggested that these seven primers could successfully amplify the *DfFPS1* gene promoter. Therefore, these new FP primers can be used for FPNI-PCR-based cloning of promoter sequences from species without reference genomes.

In this study, the promoters of the *DfFPS* genes exhibited high transcriptional activity in the stomata, which has not been reported in previous studies. Moreover, we found that the transcriptional activity of truncated *DfFPS2* promoters was lower under various conditions, whereas the transcriptional activity region of the *DfFPS1* gene promoters was mainly concentrated between the *proFPS1-Full* and *proFPS1-Δ1* regions. In future studies, this sequence can be used for yeast one-hybrid screen library to identify transcription factors regulating *DfFPS1* and to screen potential transcription factors regulating terpenoid metabolism and plant stress resistance.

## Conclusions

The *DfFPS1* and *DfFPS2* genes were screened from the transcriptome database of *D. fragrans*. The CDS of DfFPS1 (1041 bp) and DfFPS2 (1065 bp) genes were cloned, and their analyses revealed that both genes contained five conserved domains that are also conserved in other ferns. The two genes were highly expressed in the leaves of gametophytes and mature sporophytes; *DfFPS1* gene may play a major role in plant growth and development. Moreover, these two genes mainly responded to MeJA treatment and high temperature and drought stress. Both *DfFPS* genes were localized in the cytoplasm and could catalyze the synthesis of FPP *in vitro*. We overexpressed *DfFPS1* and *DfFPS2* in tobacco plants and observed that the two genes did not exhibit a significant effect on plant growth and development, but they increased the yield of terpenoids such as neophytadiene, manool, and geranyl linalool. Moreover, we observed that the transgenic tobacco plants exhibited resistance to high temperature and drought.

The FPNI-PCR was used for chromosome stepping, and the method was optimized to amplify the promoter sequences of *DfFPS1* and *DfFPS2*. Moreover, both promoters exhibited high transcriptional activity in the stomata. Meanwhile both promoters responded to MeJA treatment and high temperature and drought stress, and the transcriptional core region of the *proDfFPS1* was the region between −943 bp and −740 bp.

## Data availability statement

The original contributions presented in the study are included in the article/**Supplementary Materials**. Further inquiries can be directed to the corresponding author.

## Author contributions

YC: conceptualization. DZ: methodology. XT: formal analysis. LC: data curation. XQ: writing—original draft preparation. CS: writing—review and editing. HW: supervision. All authors contributed to the article and approved the submitted version.

## Supplementary material

Supplementary Figure 1
**(A)**: *DfFPS1* amplicon; **(B)**: *DfFPS2* amplicon

Supplementary Figure 2 Sequence analysis of DfFPS and AtFPS proteins.

Supplementary Figure 3 Expression and purification of DfFPS proteins. **(A)** Expression of DfFPS1 protein. *Note*. M: marker; 1: BL21 empty bacteria; 2: pET32a empty vector; 3: pET32a-DfFPS1 (not induced); 4: pET32a-DfFPS1 (0.5 mM IPTG; precipitate); 5: pET32a-DfFPS1 (0.5 mM IPTG; supernatant); 6: pET32a-DfFPS1 (1.0 mM IPTG; precipitate); 7: pET32a-DfFPS1 (1.0 mM IPTG; supernatant); 8: pET32a-DfFPS1 (1.5 mM IPTG; precipitate); 9: pET32a-DfFPS1 (1.5 mM IPTG; supernatant). **(B)** Expression of DfFPS2 protein. *Note.* M: marker; 1: BL21 empty bacteria; 2: pET32a empty vector; 3: pET32a-DfFPS2 (not induced); 4: pET32a-DfFPS2 (0.5 mM IPTG; precipitate); 5: pET32a-DfFPS2 (0.5 mM IPTG; supernatant); 6: pET32a-DfFPS2 (1.0 mM IPTG; precipitate); 7: pET32a-DfFPS2 (1.0 mM IPTG; supernatant); 8: pET32a-DfFPS2 (1.5 mM IPTG; precipitate); 9: pET32a-DfFPS2 (1.5 mM IPTG; supernatant). **(C)** Purification of DfFPS1 protein. *Note*. M: marker; 1: pET32a-DfFPS1 (not induced); 2: pET32a-DfFPS1 (1.5 mM IPTG; supernatant); 3: binding buffer (flow); 4: wash buffer (flow); 5: elution buffer (flow). **(D)** Purification of DfFPS1 protein. *Note.* M: marker; 1: pET32a-DfFPS2 (not induced); 2: pET32a-DfFPS2 (1.0 mM IPTG; supernatant); 3: binding buffer (flow); 4: wash buffer (flow); 5: elution buffer (flow).

Supplementary Figure 4 Identification of transgenic plants. **(A)** PCR analysis of *DfFPS1*-overexpressing tobacco plants. *Note.* M: marker; −: negative control; +: positive control; WT: wild type; VC: vector control; 1–10: *35S::DfFPS1*-resistant plants. **(B)** PCR analysis of *DfFPS2*-overexpressing tobacco. *Note.* M: marker; −: negative control; +: positive control; WT: wild type; VC: vector control; 1–10: 35S::*DfFPS2*-resistant plants.

Supplementary Figure 5 Metabolite analysis of wild-type and transgenic tobacco leaves using GC-MS. **(A)** Wild-type tobacco; **(B)** *35S::DfFPS1*; **(C)** *35S::DfFPS2*. 1: nicotine; 2: neophytadiene; 3: *n*-docosanol; 4: Cis-7,10,13,16,19-docosapentaenoic acid methyl ester; 5: manool; 6: geranyl linalool; 7: eicosapentaenoic acid ethyl ester.

Supplementary Figure 6 NBT and DAB staining of wild-type and transgenic tobacco leaves (*35S::DfFPS1-9* and *35S::DfFPS2-6*) under different types of abiotic stress.

Supplementary Figure 7 Amplify the promoter sequence of the two *DfFPS* genes. **(A)** Gel electrophoresis image of *DfFPS1* promoter amplified using FPNI-PCR; **(B)** Gel electrophoresis image of *DfFPS2* promoter amplified using FPNI-PCR; **(C)** *DfFPS1* promoter amplified using (FP10-16) primers.
