# Supplementary figures and images for: Functional characterization and transcriptional activity analysis of Dryopteris fragrans farnesyl diphosphate synthase genes
Source: Front Plant Sci. 2023 Mar 24;14:1105240. doi: 10.3389/fpls.2023.1105240 (PMC10079908; doi:10.3389/fpls.2023.1105240)

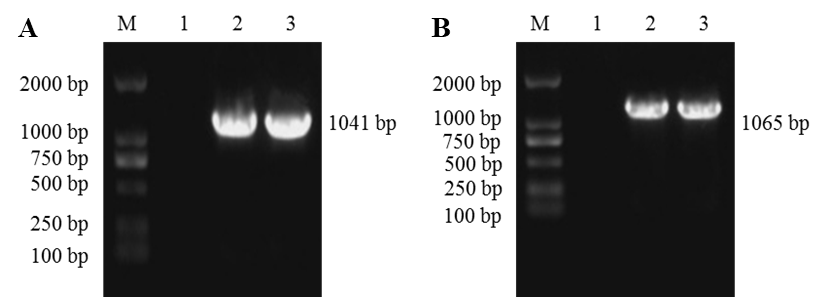

Supplement: Supplementary Figure 1 — (A): DfFPS1 amplicon; (B): DfFPS2 amplicon [file Image_1.tif]

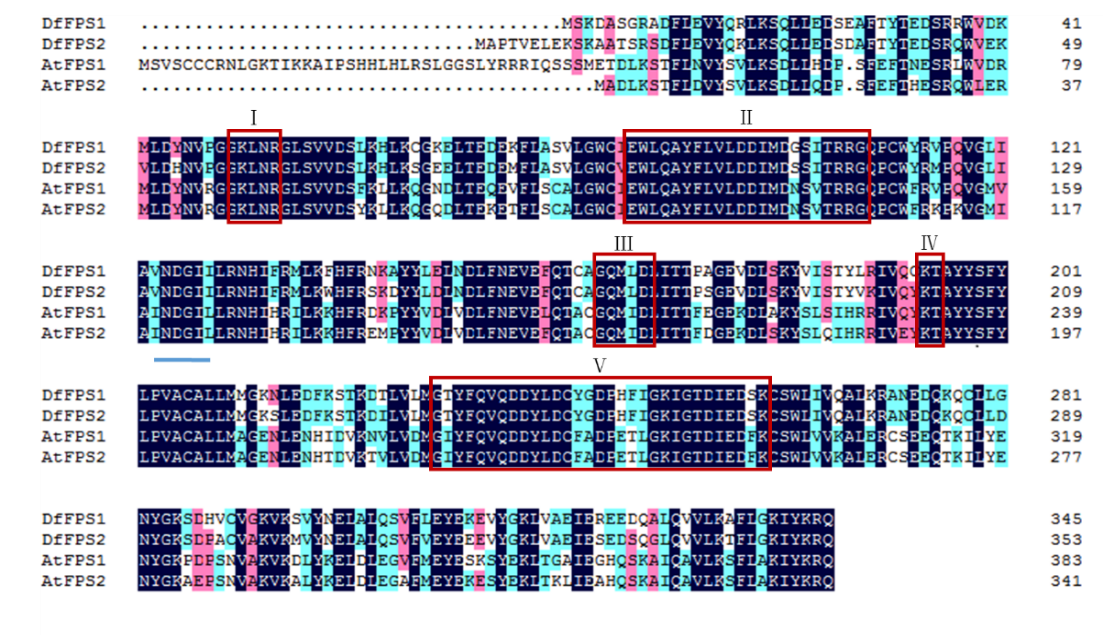

Supplement: Supplementary Figure 2 — Sequence analysis of DfFPS and AtFPS proteins. [file Image_2.tif]

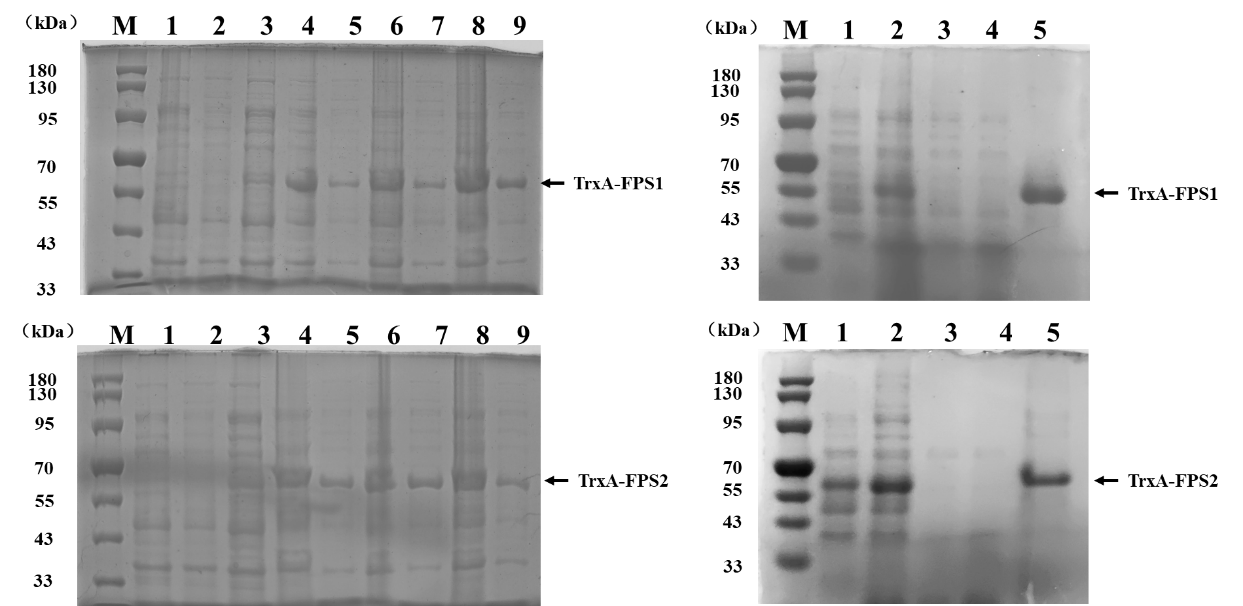

Supplement: Supplementary Figure 3 — Expression and purification of DfFPS proteins. (A) Expression of DfFPS1 protein. Note. M: marker; 1: BL21 empty bacteria; 2: pET32a empty vector; 3: pET32a-DfFPS1 (not induced); 4: pET32a-DfFPS1 (0.5 mM IPTG; precipitate); 5: pET32a-DfFPS1 (0.5 mM IPTG; supernatant); 6: pET32a-DfFPS1 (1.0 mM IPTG; precipitate); 7: pET32a-DfFPS1 (1.0 mM IPTG; supernatant); 8: pET32a-DfFPS1 (1.5 mM IPTG; precipitate); 9: pET32a-DfFPS1 (1.5 mM IPTG; supernatant). (B) Expression of DfFPS2 protein. Note. M: marker; 1: BL21 empty bacteria; 2: pET32a empty vector; 3: pET32a-DfFPS2 (not induced); 4: pET32a-DfFPS2 (0.5 mM IPTG; precipitate); 5: pET32a-DfFPS2 (0.5 mM IPTG; supernatant); 6: pET32a-DfFPS2 (1.0 mM IPTG; precipitate); 7: pET32a-DfFPS2 (1.0 mM IPTG; supernatant); 8: pET32a-DfFPS2 (1.5 mM IPTG; precipitate); 9: pET32a-DfFPS2 (1.5 mM IPTG; supernatant). (C) Purification of DfFPS1 protein. Note. M: marker; 1: pET32a-DfFPS1 (not induced); 2: pET32a-DfFPS1 (1.5 mM IPTG; supernatant); 3: binding buffer (flow); 4: wash buffer (flow); 5: elution buffer (flow). (D) Purification of DfFPS1 protein. Note. M: marker; 1: pET32a-DfFPS2 (not induced); 2: pET32a-DfFPS2 (1.0 mM IPTG; supernatant); 3: binding buffer (flow); 4: wash buffer (flow); 5: elution buffer (flow). [file Image_3.tif]

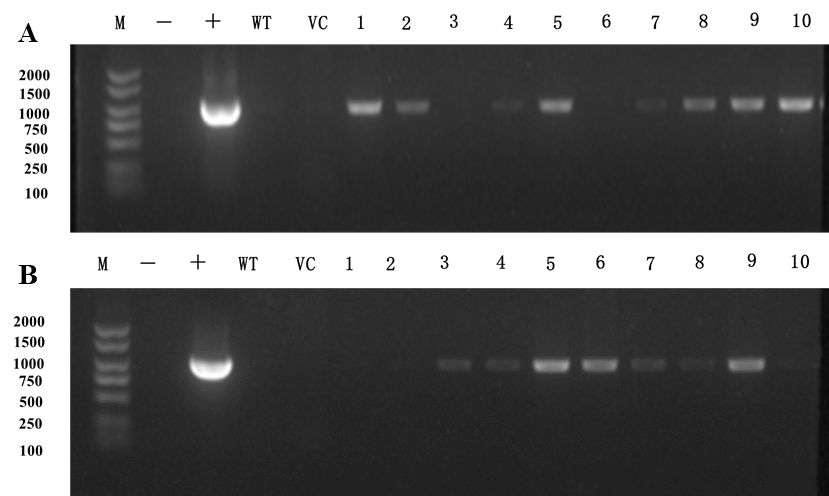

Supplement: Supplementary Figure 4 — Identification of transgenic plants. (A) PCR analysis of DfFPS1-overexpressing tobacco plants. Note. M: marker; −: negative control; +: positive control; WT: wild type; VC: vector control; 1–10: 35S::DfFPS1-resistant plants. (B) PCR analysis of DfFPS2-overexpressing tobacco. Note. M: marker; −: negative control; +: positive control; WT: wild type; VC: vector control; 1–10: 35S::DfFPS2-resistant plants. [file Image_4.tif]

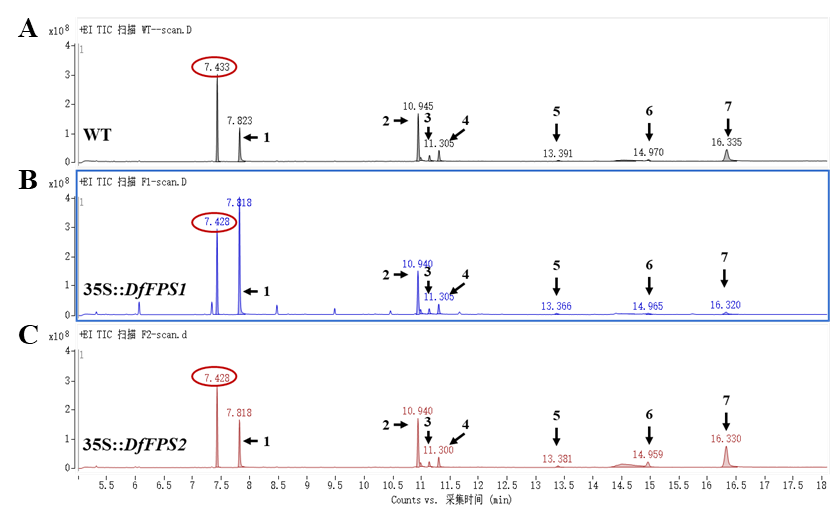

Supplement: Supplementary Figure 5 — Metabolite analysis of wild-type and transgenic tobacco leaves using GC-MS. (A) Wild-type tobacco; (B) 35S::DfFPS1; (C) 35S::DfFPS2. 1: nicotine; 2: neophytadiene; 3: n-docosanol; 4: Cis-7,10,13,16,19-docosapentaenoic acid methyl ester; 5: manool; 6: geranyl linalool; 7: eicosapentaenoic acid ethyl ester. [file Image_5.tif]

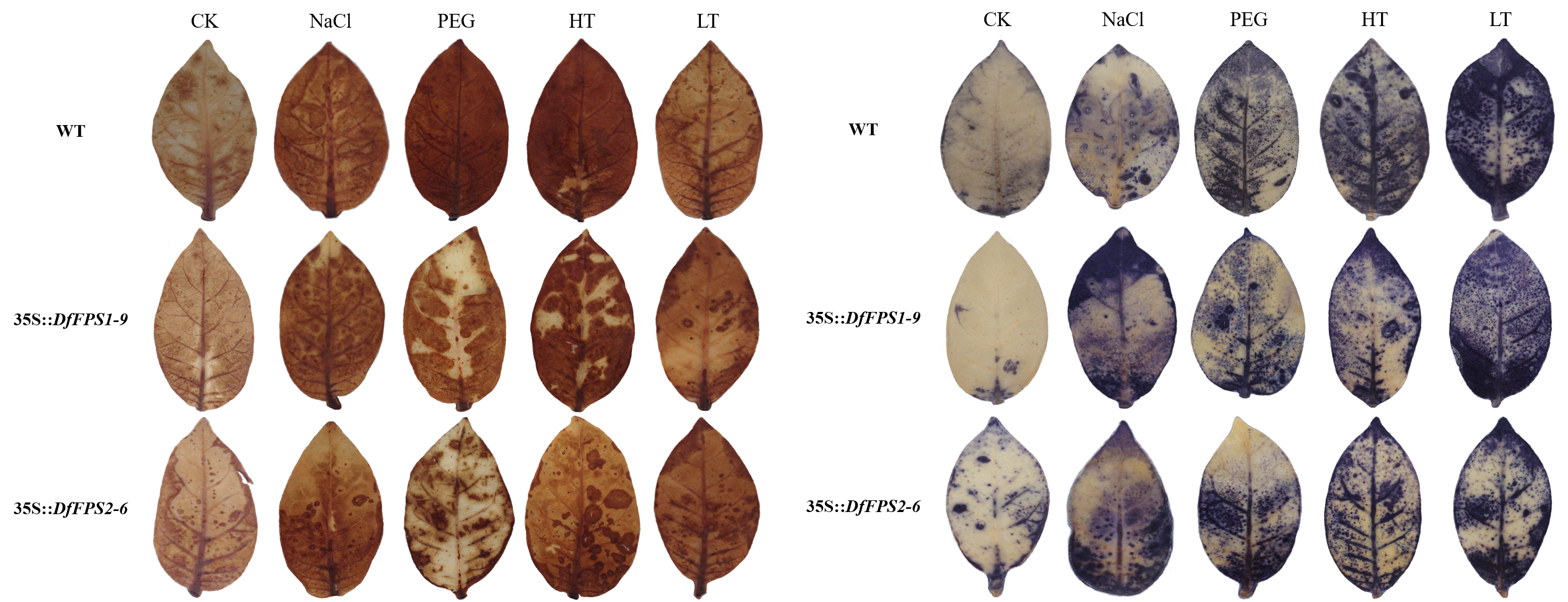

Supplement: Supplementary Figure 6 — NBT and DAB staining of wild-type and transgenic tobacco leaves (35S::DfFPS1-9 and 35S::DfFPS2-6) under different types of abiotic stress. [file Image_6.tif]

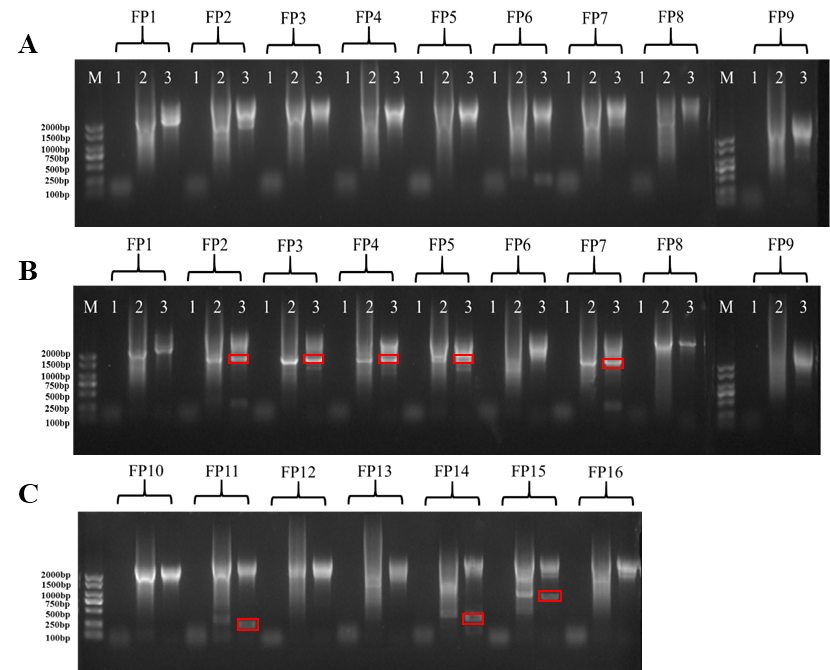

Supplement: Supplementary Figure 7 — Amplify the promoter sequence of the two DfFPS genes. (A) Gel electrophoresis image of DfFPS1 promoter amplified using FPNI-PCR; (B) Gel electrophoresis image of DfFPS2 promoter amplified using FPNI-PCR; (C) DfFPS1 promoter amplified using (FP10-16) primers. [file Image_7.tif]
