# Supplementary material for: Functional characterization and transcriptional activity analysis of Dryopteris fragrans farnesyl diphosphate synthase genes
Source: Front Plant Sci. 2023 Mar 24;14:1105240. doi: 10.3389/fpls.2023.1105240 (PMC10079908; doi:10.3389/fpls.2023.1105240)
Supplement: Supplementary file 8 [file Table_1.docx]

**Table S1** FPS sequences used in this study

| No. | Protein ID | Gene name | *Species* |
| --- | --- | --- | --- |
| 1 | AFO53556.1 | HsFPS | *Huperzia serrata* |
| 2 | XP_024363271.1 | PpFPS | *Physcomitrium patens* |
| 3 | KAG0622987.1 | CpFPS | *Ceratodon purpureus* |
| 4 | AFO53558.1 | PcFPS | *Phlegmariurus carinatus* |
| 5 | AIL88642.1 | PmFPS | *Pinus massoniana* |
| 6 | QHZ00912.1 | TcFPS | *Taiwania cryptomerioides* |
| 7 | ACA21460.1 | PaFPS | *Picea abies* |
| 8 | QBS32940.1 | PtFPS | *Pinus taeda* |
| 9 | AAR27053.1 | GbFPS | *Ginkgo biloba* |
| 10 | AAS19931.1 | TxmFPS | *Taxus x media* |
| 11 | ADV03674.1 | AoFPS | *Alisma orientale* |
| 12 | AFX68799.1 | DoFPS | *Dendrobium officinale* |
| 13 | AFV51839.1 | TaFPS | *Triticum aestivum* |
| 14 | NP_001105039.1 | ZmFPS | *Zea mays* |
| 15 | XP_008792798.1 | PdFPS | *Phoenix dactylifera* |
| 16 | ADJ67472.1 | AraFPS | *Artemisia annua* |
| 17 | AAV58896.1 | CaFPS | *Centella asiatica* |
| 18 | AEY77151 .1 | EsFPS | *Eleutherococcus senticosus* |
| 19 | ACN63187.1 | EkFPS | *Euphorbia pekinensis* |
| 20 | ADE18770.1 | GuFPS | *Glycyrrhiza uralensis* |
| 21 | AAM98379.1 | HbFPS | *Hevea brasiliensis* |
| 22 | BAB40665.1 | HlFPS | *Humulus lupulus* |
| 23 | ADC32809.1 | MsFPS | *Medicago sativa* |
| 24 | AF384040.1 | MxpFPS | *Mentha*×*piperita* |
| 25 | AAY87903.1 | PgFPS | *Panax ginseng* |
| 26 | AAY53905.1 | PnFPS | *Panax notoginseng* |
| 27 | ADJ68004.1 | PqFPS | *Panax quinquefolius* |
| 28 | ABV08819.1 | SmFPS | *Salvia miltiorrhiza* |
| 29 | ADO87007.1 | SaFPS | *Santalum album* |
| 30 | NP_001234068.1 | SlFPS | *Solanum lycopersicum* |
| 31 | AAX76910.1 | VvFPS | *Vitis vinifera* |
| 32 | AHZ21075.1 | TksFPS | *Taraxacum kok-saghyz* |
| 33 | AHM22931.1 | NtFPS | *Nicotiana tabacum* |
| 34 | NP_199588.1.1 | AtFPS1 | *Arabidopsis thaliana* |
| 35 | NP_193452.1.1 | AtFPS2 | *Arabidopsis thaliana* |
| 36 | AAN62522.1 | EuFPS | *Eucommia ulmoides* |
