# Supplementary material for: Functional characterization and transcriptional activity analysis of Dryopteris fragrans farnesyl diphosphate synthase genes
Source: Front Plant Sci. 2023 Mar 24;14:1105240. doi: 10.3389/fpls.2023.1105240 (PMC10079908; doi:10.3389/fpls.2023.1105240)
Supplement: Supplementary file 9 [file Table_2.docx]

**Table S2** Primers for qPCR

| Gene | Primer name | Primer sequence |
| --- | --- | --- |
| *Df18S* | 18S-q-F | 5′-GCTTTCGCAGTAGTTCGTCTTC-3′ |
|  | 18S-q-R | 5′-TGGTCCTATTATGTTGGTCTTCGG-3′ |
| *DfFPS1* | FPS1-q-F | 5′-TTTACCTTCCGGTTGCTTGT-3′ |
|  | FPS1-q-R | 5′-TGAACAATCAGCCAAGAGCA-3′ |
| *DfFPS2* | FPS2-q-F | 5′-TCCAGTTGCATGTGCTCTTT-3′ |
|  | FPS2-q-R | 5′-ACAATCAGCCAGGAGCATTT-3′ |
