# Supplementary material for: Functional characterization and transcriptional activity analysis of Dryopteris fragrans farnesyl diphosphate synthase genes
Source: Front Plant Sci. 2023 Mar 24;14:1105240. doi: 10.3389/fpls.2023.1105240 (PMC10079908; doi:10.3389/fpls.2023.1105240)
Supplement: Supplementary file 10 [file Table_3.docx]

**Table S3** Primers used for FPNI-PCR

| Primer name | Primer sequence | Purpose |
| --- | --- | --- |
| FP1 | 5′-GTAATACGACTCACTATAGGGCACGCGTGGT  NTCGA STWTS GWGTT-3′ | 1^st^ PCR |
| FP2 | 5′-GTAATACGACTCACTATAGGGCACGCGTGGT  NGTCG ASWGA NAWGAA-3′ | 1^st^ PCR |
| FP3 | 5′-GTAATACGACTCACTATAGGGCACGCGTGGT  WGTGN AGWAN CANAGA-3′ | 1^st^ PCR |
| FP4 | 5′-GTAATACGACTCACTATAGGGCACGCGTGGT  AGWGN AGWAN CAWAGG-3′ | 1^st^ PCR |
| FP5 | 5′-GTAATACGACTCACTATAGGGCACGCGTGGT  NGTAW AASGT NTSCA A-3′ | 1^st^ PCR |
| FP6 | 5′-GTAATACGACTCACTATAGGGCACGCGTGGT  NGACG ASWGA NAWGAC-3′ | 1^st^ PCR |
| FP7 | 5′-GTAATACGACTCACTATAGGGCACGCGTGGT  NGACG ASWGA NAWGAA-3′ | 1^st^ PCR |
| FP8 | 5′-GTAATACGACTCACTATAGGGCACGCGTGGT  GTNCG ASWCA NAWGTT-3′ | 1^st^ PCR |
| FP9 | 5′-GTAATACGACTCACTATAGGGCACGCGTGGT  NCAGC TWSCT NTSCTT-3′ | 1^st^ PCR |
| FSP1 | 5′-GTAATACGACTCACTATAGGGC-3′ | 2^nd^ PCR |
| FSP2 | 5′-ACTATAGGGCACGCGTGGT-3′ | 3^rd^ PCR |
