# Supplementary material for: Functional characterization and transcriptional activity analysis of Dryopteris fragrans farnesyl diphosphate synthase genes
Source: Front Plant Sci. 2023 Mar 24;14:1105240. doi: 10.3389/fpls.2023.1105240 (PMC10079908; doi:10.3389/fpls.2023.1105240)
Supplement: Supplementary file 11 [file Table_4.docx]

**Table S4** Gene specific primers for *DfFPS1* and *DfFPS2*

| Primer name | Primer sequence | Purpose |
| --- | --- | --- |
| DfFPS1-SP1 | 5′-TAGAAAGTCGGCACGGCCTGATG-3′ | 1^st^ PCR |
| DfFPS1-SP2 | 5′-TGGGCTCGAAACGCTTCAAGAAGA-3′ | 2^nd^ PCR |
| DfFPS1-SP3 | 5′-TGCGCGAATGGAGATCACCTGACAC-3′ | 3^rd^ PCR |
| DfFPS2-SP1 | 5′-GGAAATCCGAACGACTTGTCGCAG-3′ | 1^st^ PCR |
| DfFPS2-SP2 | 5′-CTCCAGTTCCACAGTAGGAGCCAT-3′ | 2^nd^ PCR |
| DfFPS2-SP3 | 5′-CAATGGAGGGCTGAAGTCGTTGCG-3′ | 3^rd^ PCR |
