# Supplementary material for: Functional characterization and transcriptional activity analysis of Dryopteris fragrans farnesyl diphosphate synthase genes
Source: Front Plant Sci. 2023 Mar 24;14:1105240. doi: 10.3389/fpls.2023.1105240 (PMC10079908; doi:10.3389/fpls.2023.1105240)
Supplement: Supplementary file 12 [file Table_5.docx]

**Table S5** Conditions for different cycles of FPNI-PCR

| PCR reaction | Cycle number | PCR condition |
| --- | --- | --- |
| 1^st^ PCR  FP (1 μM)  GSP (0.2 μM) | 1  2  1  6  1 | 95℃: 90 s  94℃: 10 s; 62℃: 30 s; 72℃: 2 min  94℃: 10 s; 25℃: 2 min; 0.2℃/s; 72℃: 2 min  94℃: 10 s; 62℃: 30 s; 72℃: 2 min  94℃: 10 s; 62℃: 30 s; 72℃: 2 min  94℃: 10 s; 44℃: 30 s; 72℃: 2 min  72℃: 5 min |
| 2^nd^ PCR  (0.5 μM) | 1  30  1 | 95℃: 90 s  94℃: 10 s; 62℃: 30 s; 72℃: 2 min  72℃: 5 min |
| 3^rd^ PCR  (0.5 μM) | 1  12  1 | 95℃: 90 s  94℃: 10 s; 62℃: 30 s; 72℃: 2 min  72℃: 5 min |
