# Supplementary material for: Functional characterization and transcriptional activity analysis of Dryopteris fragrans farnesyl diphosphate synthase genes
Source: Front Plant Sci. 2023 Mar 24;14:1105240. doi: 10.3389/fpls.2023.1105240 (PMC10079908; doi:10.3389/fpls.2023.1105240)
Supplement: Supplementary file 13 [file Table_6.docx]

**Table S6** Primers used for amplifying truncated *DfFS1* and *DfPS2* promoters

| Gene | Primer name | Primer sequence |
| --- | --- | --- |
| *DfFPS1* | pF1-full-F | 5′-CATGATTACGCCAAGCTTGCTGGCCTCAGGTGCTAT-3′ |
|  | pF1-∆1-F | 5′-CATGATTACGCCAAGCTTGGCTAGATTATTGACTGGCAA-3′ |
|  | pF1-∆2-F | 5′-CATGATTACGCCAAGCTTCAGATTTCCATCCCCTACA-3′ |
|  | pF1-R | 5′-CTGACCACCCGGGGATCCTGATGCATCCTTGGACAT-3′ |
| *DfFPS2* | pF2-full-F | 5′-CATGATTACGCCAAGCTTAGCAACGATAGGCATGC-3′ |
|  | pF2-∆1-F | 5′-CATGATTACGCCAAGCTTGATTTGCTCCAGAGGGAAAC-3′ |
|  | pF2-∆2-F | 5′-CATGATTACGCCAAGCTTATCTTGGGTCAGAGAGG-3′ |
|  | pF2-∆3-F | 5′-CATGATTACGCCAAGCTTGCATTAATGCAGAATGGC-3′ |
|  | pF2-∆4-F | 5′-CATGATTACGCCAAGCTTATTGGCGAGCATTATCTC-3′ |
|  | pF2-R | 5′-CTGACCACCCGGGGATCCAGGAGCCATTATCTTCTAAGC-3′ |
